# The Neural Correlates of Conflict Detection and Resolution During Multiword Lexical Selection: Evidence from Bilinguals and Monolinguals

**DOI:** 10.3390/brainsci9050110

**Published:** 2019-05-14

**Authors:** Manuel F. Pulido, Paola E. Dussias

**Affiliations:** Department of Spanish, Italian and Portuguese, The Pennsylvania State University, University Park, PA 16802, USA; pdussias@psu.edu

**Keywords:** lexical selection, conflict, multiword units, collocations, bilingualism, ERPs

## Abstract

Previous studies have identified the Event Related Potential (ERP) components of conflict detection and resolution mechanisms in tasks requiring lexical selection at the individual word level. We investigated the brain potentials associated with these mechanisms in a lexical selection task based on multiword units made up of verb–noun combinations (e.g., *eat breakfast, skip school*). Native and non-native English speakers were asked to select a familiarized target verb–noun sequence (*eat breakfast*) between two choices. Trials were low-conflict, with only one plausible candidate (e.g., *eat – shoot – breakfast*) or high-conflict, with two plausible verbs (e.g., *eat – skip – breakfast*). Following the presentation of the noun, native English speakers showed a biphasic process of selection, with a conflict-detection centro-parietal negativity between 500 and 600 ms (N_inc_), followed by a right frontal effect (RFE) between 600 and 800 ms preceding responses. Late Spanish–English bilinguals showed a similar but more sustained and more widespread effect. Additionally, brain activity was only significantly correlated with performance in native speakers. Results suggest largely similar basic mechanisms, but also that different resources and strategies are engaged by non-native speakers when resolving conflict in the weaker language, with a greater focus on individual words than on multiword units.

## 1. Introduction

Previous research has shown that bilinguals experience lexical competition from cross-language activation [1,2,3], even when the other language is not in use. Because lexical selection involves detecting conflict between candidates concurrently available for selection, understanding the process of lexical selection requires investigating the conflict detection and resolution mechanisms involved. Increased interest on this question is evidenced by the large number of studies that have focused on the consequences that resolving conflict in lexical selection has in bilinguals’ linguistic and cognitive performance [4,5,6,7,8]. However, research on the specific mechanisms involved in detecting and resolving conflict in actual language usage is surprisingly lacking. More specifically, while some paradigms such as Stroop have been used to investigate lexical selection at the individual word level, there is a notorious absence of work investigating how lexical selection occurs in units greater than a single word (with the exception of studies investigating selection of the determiner given a noun, e.g., [9,10,11,12]). The classic Stroop task is an excellent paradigm to explore the cognitive mechanisms involved in conflict detection and resolution during lexical selection, given its simplicity in design. Moreover, the mechanisms elicited by the task are assumed to be domain-general mechanisms, common to other cognitive tasks (see [13] for a review of ERP findings on Flanker and Stroop), and directly related to selection in language use [14,15,16]. But while the simplicity of the Stroop task has provided a valuable tool into the mechanisms of conflict detection, it is obviously not without its limitations. Perhaps most preeminent is the fact that word selection in actual linguistic performance does not occur in the form of independent lexical choices; rather, choices are made to connect words with other words to form phrases that themselves are then nested into sentences, and these in turn into broader discourse. Critically, words to be selected must be an acceptable lexical choice based on the surrounding context. Put simply, only words that “fit” the syntactic and semantic context need to be selected.

In this study, we used a novel Stroop-like task to examine lexical selection in multiword units, where a word to be selected is strongly associated with an ensuing word because of their tendency to appear together. We recorded electroencephalogram (EEG) while native English speakers and Spanish–English bilinguals were presented with critical trials containing either conflicting or unrelated (non-conflicting) pairs of verbs; participants selected the target verb based on the presence of a noun. By comparing trials in which only one verb is a plausible candidate (Example 1) to trials in which two verbs compete for selection (Example 2), we are able to explore the neural correlates of conflict detection during lexical selection in multiword units.
(1) *eat – shoot*    *breakfast*   familiarized target verb: *EAT*(2) *eat – skip*   *breakfast*


The paradigm used here to examine lexical selection does not rely on the same cross-modal cues present in studies that have employed the Stroop paradigm (e.g., word-color) but instead relies on lexical cues; this allows us to investigate the same basic conflict-detection mechanisms widely investigated in the behavioral and ERP literature on semantic interference based on the Stroop effect.

### 1.1. Background

#### 1.1.1. A Multiword Units Approach to Lexical Access and Selection in the L1 and the L2

The importance of making acceptable lexical choices is an aspect that non-native speakers become acutely aware of in any occasion in which knowledge from the native language (L1) is found to not be directly applicable to the other language. For instance, while an individual can be said to *skip breakfast* in English, its idiomatic Spanish equivalent *saltarse el desayuno* “jump the breakfast”, if literally translated, would present a native English speaker with a very unusual phrase. Multiword units like these are highly frequent in everyday language usage, with some estimates suggesting that approximately 50% of the choices used by speakers are part of conventional expressions [17]. A growing number of studies supports the view that frequent word combinations are stored and retrieved as units. For instance, familiar binomials such as “black and white” or “bride and groom” are processed faster than control sequences composed of words matched in individual frequency but not in co-occurrence frequency [18,19]. Most compellingly, differences are found when the same words are presented in an unconventional order, e.g., the unconventional “white and black” relative to the familiar “black and white.” The literature thus suggests that conventional word choices like *skip breakfast* are not retrieved as independent items, i.e., *skip + breakfast,* but rather as pre-stored connections between words, or multiword units [20,21,22,23]. Multiword units offer valuable insight into the role of automatic lexical activation as based on the strength of experience-based lexical associations. Familiar multiword units come online when a given target is accessed, i.e., *breakfast,* which then may activate common collocates, e.g., *eat* but also non-target candidates such as *skip, serve,* or *prepare.*

In order to investigate conflict detection that results from interference between lexical candidates, one must first understand when interference during selection of an item in a given context may arise. In the presence of two candidates (*eat* and *shoot*) in a context in which the noun *breakfast* follows, the pre-existing association between *eat* and *breakfast* will more highly activate *eat*, favoring its selection. A lack of (or very weak) association between *shoot* and *breakfast* will result in little interference from this non-target candidate.

There is a debate in the literature surrounding how lexical selection takes place, in order to account for mixed findings in studies on lexical selection (see, e.g., the Response Exclusion Hypothesis (REH) [24]; or the Competition Hypothesis [25]). We acknowledge this debate and clarify that, in this paper, our intent is not to adjudicate between these accounts. Instead, we base the logic of the experiment on theories of bilingual lexical selection, where there is agreement that competition exists between languages at multiple levels (e.g., [26,27,28,29]).

Monolingual speakers would be thought to experience interference stemming from within-language spreading activation that needs to be resolved. However, this problem is exacerbated in bilinguals, who when using another language also experience cross-language activation [1,2,3]. In the current study, we aim to examine conflict that arises from the activation of lexical units greater than single words. That is, we build on the finding that native and non-native speakers store representations of multiword units (e.g., “black and white”, “bride and groom” or “to eat breakfast”; [18,19]) to better understand the complexity of conflict during bilingual lexical selection. The consequence of automatic spreading activation in bilinguals is that competition will arise within and across languages. In non-balanced bilinguals (e.g., L1 Spanish–L2 English bilinguals), an asymmetry in competition is expected to occur when lexical associations depend on knowledge of L2 multiword units that are incongruent with the native language (e.g., *skip* – *saltarse* “jump” *breakfast*).

Previous studies have shown that the processing of L1–L2 incongruent verb–noun phrases is slower relative to L1–L2 congruent controls [30,31,32,33]. The evidence suggests that, even after they have been learned, incongruent multiword units in the L2 are less entrenched and more weakly represented in proficient L2 speakers, relative to multiword units that also exist in the L1. However, this question has so far not been explored in the processing of verb–noun (V-N) phrases employing neurophysiological measures.

An exception to the general absence of research focusing on lexical selection in multiword units is made by a number of studies examining selection of the determiner. These studies have usually employed the picture–word interference paradigm [11,12,32,33]. The common finding is that there are slower naming latencies when the determiner of the distractor noun is incongruent with the determiner of the target, consistent with a cost due to conflict during selection. Dhooge et al. [9] recently used ERPs to investigate the timing of determiner selection. In their experiment, participants were required to name a picture or withhold their response based on the determiner for the noun, using a paradigm that capitalized on the go-nogo N200 component. The results suggested that distractor nouns with an incongruent gender interfered during selection of the target noun. But because the manipulation was designed to elicit differences in the go-nogo N200, which reflects response inhibition, no conclusions about conflict detection during selection can be made.

As illustrated by the extant evidence, variations in the timing of conflict-inducing elements and task demands mean that non-negligible differences should be expected in the timing of these processes. Investigating this question with neurophysiological measures is important because it will allow us to determine (1) the timing of conflict detection during multiword units lexical selection in native speakers. Further, the present study will explore two important questions concerning conflict detection in bilinguals: (2) whether the timing of conflict detection during selection in multiword units lexical will differ in L2 speakers, relative to monolingual speakers; and (3) whether conflict will differ in magnitude when distractors are based on knowledge of L1–L2 incongruent multiword units.

In the remainder of this section, we briefly review the relevant ERP literature on components related to conflict detection; we then describe the goals and predictions for the current study regarding the time-course of lexical selection in multiword units by taking direction from the studies reviewed here. Results for two groups of speakers are then presented that examine conflict detection during lexical selection: the first group included native English speakers to address question (1). A second group included late Spanish–English bilinguals, and allowed us to address questions (2) and (3).

#### 1.1.2. Conflict-Related ERP Components

In order to examine the issue at hand, we review the literature on related paradigms that has explored ERP components associated with conflict detection. Since the question of conflict detection has not been explored in connection with lexical selection in multiword units, we turn to the literature on conflict detection in widely-researched cognitive tasks. As this body of research demonstrates, while many tasks rely on similar basic domain-general mechanisms, the particulars of each paradigm critically affect the timing of conflict detection. Here, we briefly review this literature, which evidences similarities and differences across experimental paradigms.

Several studies have identified the electrophysiological correlates of conflict detection using both linguistic and non-linguistic tasks [13,34,35,36]. While tasks such as Flanker and Stroop differ in important ways, they are also believed to rely on the same basic conflict detection and resolution mechanisms. Flanker is a non-linguistic task, in which participants must indicate the direction of an arrow, typically by pressing a button (and ignore distractor arrows in incongruent trials). Stroop is a psycholinguistic task in which participants must retrieve the name of a color and ignore other related information. Typically, participants have to name the font color in congruent (e.g., the word BLUE printed in blue font) and incongruent trials (e.g., the word BLUE printed in red). While both tasks may rely on the same domain-general mechanisms, there are important differences across both paradigms that affect the timing of components in ERP studies. For instance, conflict detection in the Flanker task is indexed by the N200 component, a negative deflection peaking at around 200 ms after stimulus presentation in incongruent trials. In the Stroop paradigm, on the other hand, conflict detection appears much later and is indexed by the N_inc_ component, peaking at around 450 ms post-stimulus presentation over the left centro-parietal scalp, and is sometimes referred to as N450 [35,36,37]. Although the scalp distribution of the N_inc_ is typically over left central-parietal electrodes [35,37,38], source localization techniques suggest that it is generated in the pre-frontal cortex by the anterior cingulate cortex (ACC) [34,39,40], which is associated with executive control. The N_inc_ is elicited after the word and color are presented at the same time in the incongruent condition relative to congruent and control trials. Given that Stroop is a linguistic task, involving semantic processing of the word and color, and that lexical access is indexed by the N400, it is only expected that conflict detection in this paradigm will be at least as slow as semantics.

A Late Positive Component (LPC), or slow positivity component, is also commonly reported in ERP studies using the Stroop paradigm. This is a positive-going wave appearing between 600 and 900 ms post-stimulus over centro-parietal regions, inverting polarity over the anterior scalp concomitantly in time. It has been suggested to be an index of response selection, on the grounds that it correlates with RT and accuracy [41]. Others, however, have proposed that the LPC reflects semantic re-activation of the word [34]. Therefore, while it appears to be associated with the generation of a response, its functions are less clear.

One important question concerns whether the N_inc_ reflects conflict detection or conflict resolution. An insightful approach to address this question is offered by experiments that have varied the stimulus onset asynchrony (SOA) by presenting color and word stimuli at different times to investigate the precise timing of color and word interference [42,43,44]. Manipulating the SOA of the word relative to the color stimulus allows for the dissociation of automatic conflict detection from the need to resolve conflict to produce a response. In a recent ERP study, Coderre et al. [42] used long SOA manipulations of −400 ms, 0 and +400 ms. Because RTs in manual Stroop studies have been shown to occur at around 500 ms, a response is already being prepared or made by the time the word is presented in the +400 SOA condition. The authors predicted that if the N_inc_ is an index of conflict resolution, it should be absent in the positive SOA after a response has been made; if it indicates conflict detection, however, it should still be elicited. The results of the study found that the N_inc_ was still present at 700–850 ms after presentation of the color and after a response was made. This was the case even in fast-response trials, in which responses had been recorded by 600 ms. The same type of SOA manipulation has provided further insight into the LPC, showing that its presence is determined by the need to execute a response. In an EEG Stroop study with short SOA manipulations, Appelbaum et al. [37] found that in the −100 ms and −200 ms conditions, the LPC appeared earlier by the same amounts of time, respectively, and was similarly delayed in the +100 SOA condition. The longer SOA used in Coderre et al. [42] revealed a shift window in the −400 ms condition, but no LPC when presentation of the word was delayed by 400 ms.

The question of how conflict detection in the Stroop task may be affected by bilingualism has been addressed in some prior research. Several ERP studies give compelling support to the idea that L2 lexical access is slower relative to the L1 (for an overview see [45]). More specific to the Stroop task, a previous study found that Chinese–English bilinguals were slower by a difference of 100 ms in their L2 [46]. The authors investigated two aspects of conflict detection hypothesized to have opposite effects in bilinguals. On the one hand, bilingual speakers should experience slower lexical access in their L2; on the other, based on research suggesting a bilingual advantage in cognitive control, bilinguals should be faster in detecting conflict. Overall, the results gave support to a delay when the task is performed in the L2, and found some evidence for a bilingual advantage modulated by L2 proficiency. Interestingly, the results from the Chinese–English bilinguals did not give support to the idea of a bilingual L2 lexical disadvantage hypothesis, by which lower L2 proficiency should result in less interference due to weaker lexical access. Rather, lower proficiency resulted in a larger interference effect in the L2 English. The authors attributed these differences to a bilingual cognitive advantage, with a lesser cost in the higher-proficiency group. Therefore, the evidence to date highlights the variety of factors specific to bilinguals that may impact performance in the Stroop paradigm.

### 1.2. The Present Study

In the current study, we aim to investigate the mechanisms of conflict detection among competing lexical choices. We take direction from the ERP and behavioral literature on paradigms involving lexical selection to explore conflict-detection mechanisms in a paradigm that involves the selection of words within phrases. Given previous findings of conflict-detection components in tasks that have been widely studied (including the Stroop and Flanker tasks), we expect a functionally similar component to be elicited when conflict arises during selection of words within multiword units. However, the time-course of conflict detection is expected to vary as a result of the demands of the task at hand. In the present study, rather than making selection contingent on modality (as in the classic Stroop), we designed a task in which the cue for selection is provided by a word that frequently co-occurs with the target. By making lexical choices dependent on the meaning of another word within a familiar multiword unit, the current study seeks to go beyond the single-word level. Here, we explore conflict detection and resolution in a scenario in which candidates have already been activated; that is, the main manipulation in this paradigm lies on creating conditions that induce conflict in selection or not, by carefully controlling the lexical candidates presented (target and non-target verbs) before the cue for selection (i.e., the noun) appears. The issue of whether potential candidates are activated simultaneously or sequentially remains, to our knowledge, largely unexplored and goes beyond the scope of this paper (but see [9,10,11,12] on a related topic concerning the activation of different determiners for a noun). Yet, our manipulation draws on the assumption that speakers activate potential candidates concurrently [1,2,3]. We are able to also control for what candidates are more strongly activated and in what order, by presenting two verbs sequentially prior to the display of the noun. To control what verb was processed first and second, the paradigm manipulated the order of presentation of the Target (T) and Distractor (D) verbs. Half of the trials presented the target right before the noun (DT order), while the other half presented the verb to be discarded before the noun (TD). Table 1 presents examples of the 2 x 2 manipulation in an experimental trial (target: *eat – breakfast*); importantly, the distractor verbs are also potential candidates for selection, as they are the verbs of foil trials (e.g., *shoot photos*). Here and throughout the rest of the paper, the target verb in each example is indicated by using capital letters. Verbs in the experiment were presented in lower case.

While we have devised a paradigm intended to create propitious conditions to elicit the type of conflict that we aim to study, we refrain from making any claims about the sequence followed during lexical access when speakers are forming or are retrieving multiword phrases. Indeed, in natural language, the order of this process may be quite variable. For example, although a verb–noun sequence is a highly common order, other structures containing the same lexical combination reverse this order to noun-verb. To illustrate, structures such as relative clauses require the speaker to produce a noun before the verb is selected (e.g., “they skipped the breakfast, which was in fact delicious” vs. “the breakfast that they skipped was in fact delicious”). The latter might be more directly comparable with selection in our paradigm.

The experiment reported below will allow us to determine the time course and scalp topography of conflict detection during selection in English multiword units, in two different populations: native speakers of English and late Spanish–English bilinguals. While both groups completed the exact same task, the conflict-inducing distractors are potential candidates based on knowledge of English alone, but not based on knowledge of Spanish multiword units. This allow us to explore conflict in lexical selection in the non-native language for Spanish–English bilinguals, and to consider the role of familiarity and entrenchment in the L2.

## 2. Materials and Methods

### 2.1. Participants

Fifty-five participants were recruited, of which thirty were native speakers of English and twenty-five were late Spanish–English bilinguals. All subjects gave their informed consent for inclusion before they participated in the study. The study was conducted in accordance with the Declaration of Helsinki, and the protocol was approved by the Internal Review Board of the Pennsylvania State University (PRAMS0004598/00034810). Participants were paid 10 US dollars per hour of participation or per the corresponding fraction of an hour. All 23 native English speakers reported limited knowledge of a foreign language in a ten-point self-rated proficiency scale (mean: 3.37; SD: 2.01). Self-reported proficiency measures were collected by administering the LEAP questionnaire (a language background questionnaire) completed by participants as part of the testing procedure described below. Four subjects were excluded because they reported being simultaneous bilingual speakers of English and another language. One Spanish–English speaker was excluded due to switched language dominance, as indicated by self-reported proficiency in each language (greater dominance in English). Five additional subjects were excluded because they did not complete the second session. The remaining participants included 23 native English speakers (17 female, 6 male) and 22 late bilinguals (12 female, 10 male). All participants were right-handed, reported normal or corrected-to-normal vision, and had no history of neurological disorders.

### 2.2. Materials

Three types of materials were created: (1) a list of 16 English multiword units (i.e., collocations) and their associated distractors; (2) materials for a familiarization phase; and (3) materials for a multiword-based lexical selection task. The latter two were based on the multiword units selected in (1). We first describe each in turn.

#### 2.2.1. English Multiword Units (Collocations)

First, two lists of eight verb–noun target combinations (V-NP) were created. One list contained the target verb–noun multiword units (e.g., *cancel [a] trip, eat breakfast*), while the second list consisted of V-NP phrases used as foils (e.g., *schedule [a] time; skip school*). We will refer to these lists as targets and foils, respectively. All verb–noun units contained a noun that was congruent across the two languages (i.e., could be translated literally). To create the manipulation that examined the effect of conflict during lexical selection, two additional lists were of the following type: one list (the plausible distractor list) included combinations of foil-verb + target-noun combinations that were plausible (*schedule [a] trip*; *skip breakfast*). The final list (the implausible distractor list) included combinations of foil-verb + target-noun combinations that were implausible (*break [a] trip; shoot breakfast*). The four lists are given in Table 2.

In order to determine the plausibility and collocational status of the V-NP combinations in each of the four lists, two measures were obtained. First, plausibility ratings were collected on a scale from 1 (implausible) to 7 (plausible) through Amazon Mechanical Turk (MTurk). For all the participants, we collected language background information and excluded participants who reported not being native speakers of English. As an additional measure, we restricted IP addresses to the US. One participant who reported being a non-native speaker of English was excluded. Average plausibility ratings were calculated for responses from 29 native English speakers. The results showed that high plausibility ratings were given to target multiword units (e.g., *eat breakfast;* mean: 6.36; SD: 0.21), and foils (e.g., *skip school;* mean: 5.43; SD: 0.63), as well as to items in the plausible distractors list (e.g., *skip breakfast*; mean: 5.91; SD: 0.72). Implausible distractors were rated low in the plausibility scale (*shoot breakfast;* mean: 1.44; SD: 0.25). Based on Bonferroni-corrected pairwise comparisons, ratings for implausible non-targets were significantly lower both from those for targets (*t*(39) = 72.86, *p* < 0.001), foils (*t*(39) = 45.49, *p* < 0.001), and plausible distractors (*t*(39) = 35.7, *p* < 0.001); also, in line with the corpus-based measures, ratings revealed that targets were preferred over (plausible) distractors (*t*(39) = 3.89, *p* < 0.01).

To measure the collocational status in the V-NP sequences, t-scores [47,48,49] were calculated using data from the Corpus of Contemporary American English (COCA, Davies, 2008). T-scores have been shown to be more resistant to inflation than other association measures, such as Mutual Information (MI) scores, and allow for comparability with previous work e.g., [24]. Word pairs with t-scores equal or greater than 2.0 are considered collocational [50]. The analyses revealed that all the target and foil items were collocational, i.e., conventional multiword units (mean: 26.82; SD: 17.73). T-scores were also calculated for the plausible and implausible distractors. All plausible distractors had t-scores of at least 5.0 (mean: 12.01; SD: 4.78), confirming their collocational status. Based on log-normalized frequencies from corpus data, target V-NP phrases were more frequent (mean: 6.3; SD: 1.15) than plausible distractors (mean: 5.05; SD: 0.76; *t*(39) = 5.94, *p* < 0.0001). Implausible distractors were in all cases non-collocational. While some observations of implausible distractors were expected due to chance, given the large size of COCA (with over 570 million words), *t*-scores had negative values in all cases, well below the conventional threshold of 2.0 (min: −25.11; max: −1.44).

One final note is in order. Because the same materials were used with both groups of speakers (i.e., native and non-native), the proportion of cognates between English and Spanish was controlled, with 50% of cognate nouns in the target and foil lists. The experimental manipulation for the bilingual group also required that items in the target list be congruent between the two languages, whereas the verbs in plausible distractors needed to be incongruent between the two languages. Language congruency was determined by the two authors who are native speakers of Spanish. Additionally, verbs were normed by collecting translation data from a group of 22 Spanish–English bilinguals (mean self-rated English proficiency on a 10-point scale: 8.75; SD: 0.78). Bilinguals were asked to translate the isolated verb of each English collocation (e.g., *eat, skip*) into Spanish and the verbs of the Spanish equivalents into English (e.g., *comer, saltarse*). The combined proportion of target responses was calculated for each collocation. Target responses for the translations of the verbs of congruent collocations averaged 94% accuracy; for incongruent collocations, accuracy was only 38%.

#### 2.2.2. Familiarization Phase

A prior familiarization phase was completed so that the target verb could be correctly identified. For each item in the target list, a carrier sentence was created that contained the target V-NP combination. All sentences contained the target V-NP phrase (e.g., *eat breakfast*), which was located towards the middle of the sentence (Example 3). Participants read sentences at their own pace.
(3) Carrier sentence:*On Sundays I usually get up later and eat breakfast in my pajamas*Target multiword unit:*eat breakfast*

To ensure that the correct target V-NP units could be remembered, participants were given a gap-filler task in which they identified the correct multiword units in the sentences they had just read. The same sentences were presented again, this time with a gap instead of the target multiword unit. Two options were presented on the screen below the sentence, on the left and right side of the screen, as illustrated in Example 4.
(4) *On Sundays I usually get up later and ____________ in my pajamas.*   *at breakfast*     *skip school*

For each item, the non-target phrase was the associated foil presented in Table 2. The correct option (left or right) was selected by pressing the ipsilateral button on a Chronos button box; sentences were presented using E-Prime 3.0 (Psychology Software Tools, Pittsburgh, PA.). Participants were advised to pay close attention to the options presented as they would need to remember them in the ensuing task.

#### 2.2.3. Multiword-Based Lexical Selection Task 

**Figure 1:**
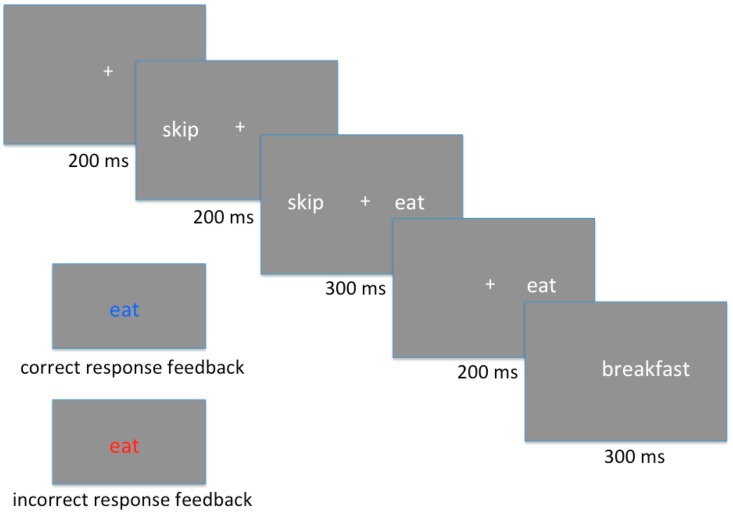
Sample trial of the Lexical Selection Task.

As described above, each multiword unit (e.g., *eat breakfast*) was matched with a plausible distractor (*skip breakfast*) and with an implausible distractor (*shoot breakfast*). In this task, participants were presented with the two verbs (target and distractor, e.g., *EAT – skip*) followed by the noun (*breakfast*). Once the noun was presented, participants were able to select the appropriate verb, based on the phrases presented during the Familiarization task (*eat* in this example). The order of presentation of target and distractor was counterbalanced. That is, *EAT – skip – breakfast* appeared for half of the trials in which *eat* was the target verb, and *skip – EAT – breakfast* in the other half. To control for the order of processing and activation of each verb (target and distractor), the two verbs were presented sequentially. At the beginning of each trial, one fixation cross appeared at the center of the screen for 200 ms. One verb then appeared to the left of the fixation cross; 200 ms later, a second verb appeared to the right of the fixation cross; both were displayed together for 300 ms, after which the verb on the left disappeared while the verb on the right remained on the screen for an additional 200 ms. The noun was then displayed for 300 ms, followed by a fixation cross that remained on the screen until a response was made. Responses were made by pressing the right or left button on the button box, to select the verb that was presented on the corresponding side of the screen. To ensure that participants were able select the target verbs, the correct verb (e.g., *eat*) was then presented as feedback for 400 ms (in blue font for correct responses, and in red font for incorrect responses). Words were displayed in font Arial size 30. The task was presented using E-Prime 3.0. A sample trial can be seen in Figure 1.

The manipulation of Plausibility of the distractor (plausible vs. implausible), altogether with the Order of presentation of Target and Distractor verbs (T-D vs. D-T), produced a 2 × 2 design. To generate a sufficient number of trials for the ERP analysis, each of the eight multiword units in each list was presented five times in each of the four conditions, producing 40 trials per condition (for a total of 320 trials). An additional ten practice trials were presented at the beginning of the task. Completing of the task took approximately 20 minutes.

### 2.3. Procedure

The study was divided into two separate sessions. In session 1, participants were consented after arriving in the lab. They then completed an English verbal fluency task to measure vocabulary knowledge and lexical access in production. In the verbal fluency task, participants were given 30 seconds to name exemplars for each of four categories presented (two animate); the score is calculated as the sum of total valid responses (i.e., no repetitions) generated for each category in the 30 seconds allotted. General English proficiency was then measured through an abridged version of the Michigan English Language Institute College English Test (MELICET, [51]) administered on a computer through Qualtrics (Qualtrics, Provo, UT), which contained a combination of 50 grammar and cloze-test items to assess overall language proficiency. Two behavioral measures of cognitive control were collected: the Stroop task [52] and the Flanker task [53]. Finally, to assess participants’ linguistic background, they completed the LEAP-Q [54].

Participants came back to the lab for the second session, and were consented again. In this session they completed the Familiarization, followed by the Multiword-based Lexical Selection Task. Each session took approximately 1 h and 30 min.

The procedure for both groups (native and non-native speakers) was identical with the exception that the non-native speakers completed an additional task. Because the verbs of foil trials (which were also used as plausible distractors) were incongruent with Spanish and specific to English, participants were administered a Familiarity Rating task at the end of the experiment. A list with all the targets and foils was presented, for which participants were asked to rate the familiarity of each verb–noun phrase multiword unit along a scale from 1 (completely unfamiliar) to 7 (completely familiar).

### 2.4. EEG Recording and Analysis

The continuous electroencephalogram (EEG) was recorded from 32 electrodes mounted in an elastic cap (EasyCap; Brain Products, GmbH) and an ActiChamp amplifier (Brain Products, GmbH) with a 24-bit analog to digital conversion (online sampling rate: 500 Hz, 0.1–100 Hz band-pass filter). Electrode impedances were kept below 5 KΩ. During recording, electrodes were referenced to the right mastoid. Grounding electrodes were mounted on the forehead and beneath the right eye. Blinks and eye-movements were measured by placing bipolar pairs of vertical (VEOG) above and below the left eye and lateral (HEOG) electrodes at the outer canthi of both eyes. Preprocessing steps and analyses were performed with MATLAB (R2016a, The Matworks, Inc.) and a combination of scripts and routines implemented in EEGLAB (v. 13.5.4b [55]) and ERPLAB (v. 5.0 [56]). Datasets were filtered offline with a 25 Hz low pass and 0.1 Hz high pass noncausal IIR Buttwerworth digital filter [57]. Segments with excessive muscular artifacts on the continuous data were manually rejected. Subsequently, an independent component analysis (ICA) was performed to extract and reject remaining ocular and muscular artifacts, following Jung et al. (2000). As indicated below (Section 4), separate ERP analyses were conducted for the groups of monolingual and bilingual speakers. The average number of independent components rejected during ICA averaged 3.3 (max: 5) in the monolingual dataset and 2.26 (max: 4) in bilinguals. Epochs ranging from −200 to 800 ms after onset of the noun were extracted from the pre-processed data. All epochs with activity exceeding ±100 μV at any electrode site were automatically removed using a peak-to-peak moving window. Less than 0.1% of data were lost due to artifact rejection in each group. Baseline correction was done relative to pre-stimulus activity. Inaccurate trials were excluded from the analysis (8.2% in monolinguals, 7% in bilinguals). Data from one bilingual participant were excluded due to excessive artifacts.

Based on the predictions, described in the next section, our analysis of the EEG data focused on two main time windows. In order to analyze differences in the processing of the noun, we analyzed the canonical N400 window (300–500 ms post-stimulus presentation [58,59]). Secondly, we predicted that conflict would emerge in trials in which a competitor had to be suppressed; the N_inc_ conflict-detection component was predicted to emerge at some point after processing of the noun and before a manual response was made. The analysis of the behavioral data determined that button presses started shortly after 800 ms, restricting the time window of potential interest to 500–800 ms. In order to objectively determine the time window of conflict detection, running *t*-tests were performed using the FDR-corrected mass univariate test of the Factorial Mass Univariate Toolbox (FMUT [60]), including Order and Plausibility as within-subjects factors. The raw data were averaged into bins of 4 ms, and analyzed from 500 to 800 ms post-stimulus. While this type of test is quite stringent and likely to underestimate a significant effect on the data, it allows to examine the time-course and scalp distribution of the effect in a more exploratory fashion. All scalp sites were considered of interest with the exception of Fp1, Fp2, T7, T8, Oz, O1, O2, which were excluded. The significant comparisons resulting from running *t*-tests were then used to determine the time window for the analysis with repeated-measures ANOVAs; these are reported in the Results section. The ANOVAs were conducted with mean amplitudes as dependent variables, and distractor Plausibility and Order as independent variables. Midline ANOVAs included Frontality as a predictor (frontal, central, parietal) and were performed on Fz, Cz and Pz. Lateralized ANOVAs with Frontality and Hemisphere (left, right) predictors were conducted on F3, F7, F8, FC1, FC2, FC5, FC6, C3, C4, CP1, CP2, CP5, CP6, P3, P4, P7, P8. Greenhouse–Geisser-corrected values are reported where appropriate. In order to better characterize the topography of significant effects, pairwise *t*-tests were performed on each scalp region (right frontal: F4, F8, FC2, FC6; left frontal: F3, F7, FC1, FC5; right posterior: CP2, CP6, P4, P8; left posterior: CP1, CP5, P3, P7). Results of post-hoc paired pairwise *t*-tests are reported with FDR-corrected *p* values.

Finally, we performed correlation tests to investigate a potential association between the amplitude of individual subjects’ ERP components and performance in lexical selection as measured by RTs and accuracy. Individual ERP measures were calculated as the difference waves of average amplitudes per condition (Plausibility, Order); behavioral scores were calculated as the difference between averages for each condition (e.g., RTs of Plausible DT – RTs of Implausible DT). Correlation tests were conducted between individual subjects’ ERP measures and RTs per condition, as well as between ERPs and accuracy.

### 2.5. Predictions

We predicted three different types of costs. One first prediction is concerned with the processing of the noun (300–500 ms time window). It is now well established by a large number of studies that semantic access is indexed by the N400 (300–500 ms time window). Based on previous behavioral [19,21,22] and EEG studies on the processing of multiword units [61,62] we predict that nouns immediately preceded by the target verb (e.g., *breakfast* in *eat breakfast*) or by a plausible distractor (*breakfast* in *skip breakfast*) would be primed (manifested in the form of an attenuated N400); however, nouns preceded by an implausible distractor (e.g., *breakfast* in *shoot breakfast*) would show a cost in processing (i.e., an enhanced N400). Our second prediction is related to conflict detection in lexical selection. A conflict-detection component (i.e., the N_inc_) will be elicited by trials in which distractors are plausible candidates for selection (e.g., *skip* in *skip-EAT breakfast*). Our prediction regarding the timing of conflict detection is that the N_inc_ component will be elicited at some point between the processing of the noun and the average response time for the selection of the verb. Consistent with the literature reviewed above, we expect this component to arise over left centroparietal electrodes. Our third prediction considers the order of the verbs (target last vs. distractor last), which is expected to affect the selection of the target. Based on a recency effect, we predict an asymmetry in cost affected by the order of presentation of target and distractor. Specifically, a greater cost in TD trials is predicted due to the need to discard the most recently (and therefore more strongly) activated candidate. We predict that the distractor-last order might in fact modulate inhibition- (e.g., N_inc_) or other selection-related components.

A final, more exploratory prediction, is that components indexing conflict detection during lexical selection might be correlated with behavioral responses. Although previous studies found no correlation between the peak latency of the N_inc_ and RTs, we hypothesized that the extent to which conflict-detection components are present might be a predictor of behavioral performance. Therefore, in the results section below we present an exploratory analysis to investigate a potential correlation between the amplitude of individual subjects’ ERP components and performance in lexical selection measured by RTs and accuracy.

#### Predictions for Non-Native Speakers

While the same predictions apply to both native and non-native speakers, there were some additional considerations concerning lexical selection in non-native speakers. First, as mentioned, potentially slower processing in L2 speakers was expected to be reflected in slower RTs as well as in delayed ERP components, relative to native speakers. However, the more critical prediction concerned the degree of interference that would be experienced by non-native speakers in trials in which the distractors presented were plausible, but only to the extent that speakers had experience of the multiword units in their L2. More specifically, we predict that the degree of interference for non-native speakers will be somewhat variable, and contingent on knowledge and familiarity with multiword units specific to English. Based on the assumption that the amount of interference would be reflected in the amplitude of conflict-related components, we predict a potential correlation between familiarity with the L2-specific multiword units and ERP difference waves.

## 3. Behavioral Results

In this section, we first present the results of the individual difference measures collected during the first session of the study, and the results of the Familiarization phase. The following subsections report on the analysis of behavioral and ERP data from the multiword-based Lexical Selection task.

The individual measures collected in session 1 served the double purpose of allowing us to characterize the tested population in detail and providing individual measures to be included in subsequent analyses. Table 3 below presents the results of the English verbal fluency task, Stroop and Flanker effects, the MELICET test, as well as linguistic background information collected through the LEAP-Q. The English verbal fluency score is the total number of valid responses. The Flanker effect was calculated by subtracting the RTs of congruent trials (all arrows pointing in the same direction) from incongruent trials (target and distractor arrows pointing in opposite directions). To obtain the Stroop effect, the RTs of baseline trials (naming the font color of a sequence of several @s) were subtracted from incongruent trials (naming the font color of, e.g., the word “BLUE” presented in red font). Stroop results are excluded for one participant due to technical failure.

Table 3 also presents the results of the multiword unit (MWU) familiarity rating task completed by bilinguals (additional details in Appendix A Table A3). The data show that non-native speakers were familiar with the expressions, but also that familiarity was lower and more variable for L1–L2 incongruent expressions, as reflected by the larger SD.

To ensure that participants had completed the Familiarization phase adequately, we examined the accuracy of responses collected on the gap-filler task (see example 3 above), which was 96% (min: 83.33, SD: 5.42) for native speakers and 93.24% (min: 83.33%, SD: 4.72) for non-native speakers. This confirmed that participants in both groups could correctly select the conventional multiword units in context. In what follows, we first report the behavioral results of the experimental trials in the Multiword-based Lexical Selection task, and then the ERP analysis. 

### 3.1. Accuracy in the Lexical Selection Task

The proportion of correct responses was calculated for each of the experimental conditions in the 2 × 2 design. To recapitulate, the manipulations involved Group (Native, Non-Native), Plausibility (plausible vs. implausible distractors), and Order (Distractor and Target vs. Target and Distractor; DT vs. TD). Table 4 presents the accuracy results by condition for both groups of speakers (“Mean Acc.”; additional details in Table A4 of the Appendix A).

The effect of Plausibility and Order was examined in both groups by performing a mixed-effect logistic regression. This analysis is ideal for binary variables such as accuracy, as it allows to analyze the unaggregated data rather than means [63]. The analyses of the behavioral data reported here and in other sections were carried out with the lme4 package [64] in R version 3.3.2 [65].

Following attempts to build maximally specified models [66], which led to convergence issues, the random effects structure was simplified [67]. Final models included random intercepts for subjects and items [68]. To control for variability in the plausibility of verbs, models also included by-subject slopes for the average normed plausibility of the distractors.

Fixed-effect factors included Group, Plausibility and Order, and their interaction, to analyze the effect of the experimental manipulation. The contribution of cognitive control was investigated by including fixed effects for the Stroop and Flanker Effect. English verbal fluency scores were also included as an index of lexical knowledge. Age was also included in order to control for potential differences across groups. All continuous variables were centered [68].

Model selection started with the mixed-effects model described. In a step-by-step backward model selection procedure, predictors were removed one by one, but were kept if the model fit was significantly improved by including the predictor (likelihood ratio test, *p* < 0.05). Parameter-specific *p* values were estimated using the normal approximation. Because Stroop results from one participant were not available, data from that subject were excluded in the initial analysis. However, the final model selected did not include Stroop, Flanker or English verbal fluency scores, as they did not significantly improve the fit of the model. The analysis was run again on the full dataset with the additional participant, and significance results and parameter estimates did not substantially differ. For completeness, we report the results of the parameters in the analysis that was performed on the full dataset.

**Figure 2:**
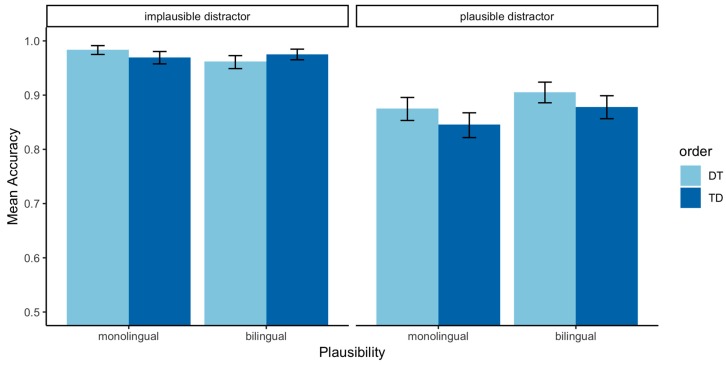
Accuracy in selection of the target verb by condition. Error bars indicate 95% confidence intervals.

The reference levels were set to native speaker for Group, implausible distractor for Plausibility, and DT for Order. The results revealed a main effect of Group (*β*: −0.96, SE: 0.34, *p <* 0.05), showing that non-natives were significantly less accurate than native speakers. There were also main effects of Plausibility (*β*: −2.37, SE: 0.31, *p <* 0.0001), with a lower accuracy when distractors were plausible relative to implausible; and Order, with more errors being produced in Target-Distractor trials (*β*: −0.65, SE: 0.32, *p <* 0.05). Additionally, two- and three-way interactions arose for Group x Plausibility (*β*: 1.31, SE: 0.38, *p <* 0.001), Group x Order (*β*: 1.01, SE: 0.42, *p <* 0.01), and Group x Plausibility x Order (*β*: −1.14, SE: 0.47, *p <* 0.05). These interactions reveal that, in bilingual speakers, implausible distractors in TD order had the opposite effect than in natives, resulting in higher accuracy (as shown by the positive *β* estimates). This is not the case in trials with plausible distractors, which have the same effect in both groups, as shown by the negative coefficient of the three-way interaction. While the interactions are somewhat complex, the pattern is clearly illustrated in Figure 2 below. The full model output is available in Appendix A (Table A1).

### 3.2. Reaction Times in the Lexical Selection Task

In order to prepare the data for RT analysis, inaccurate responses were first removed. Trials with RTs outside the absolute thresholds of 300–4000 ms were also excluded from the analysis. RTs were z-score normalized, and outliers were then removed for each participant based on their individual median absolute deviation (MAD). The MAD method is a more robust measure for outlier removal than standard deviations, given that the latter are susceptible to be distorted by observations that strongly deviate from the mean [69] (pp. 48–50). Trials above or below three absolute deviations from the median were excluded. Table 5 presents a summary of the mean RTs by condition for each group of speakers.

A linear mixed-effects regression analysis was performed on the normalized RTs. Models included the same mixed-effects structure described for accuracy data, and were built and selected using the same procedures as in the logistic regression models. The model selected contained fixed-effects for Group, Plausibility and Order. Their interactions as well as the other behavioral measures of individual differences considered (English verbal fluency, Stroop and Flanker) did not improve the model fit and were not part of the final model.

**Figure 3:**
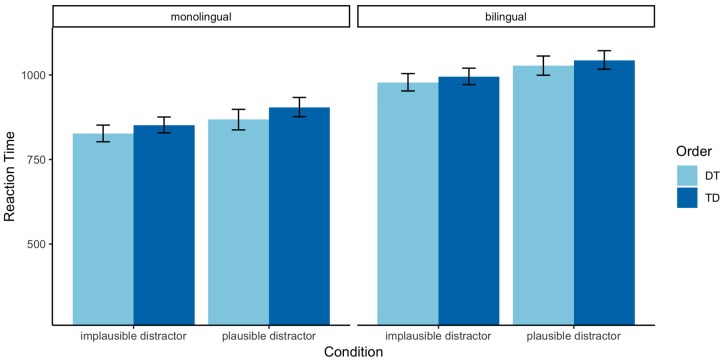
RTs for selection of the target verb by Group. The figure shows the RTs of trials with correct responses. Error bars indicate 95% confidence intervals.

The main effect of Group confirmed that Spanish–English bilingual speakers were significantly slower than native English speakers (*β*: 0.39, SE: 016, *p <* 0.05). In line with the accuracy results, the analysis revealed a main effect of Plausibility, resulting in longer RTs in trials with plausible distractors (*β*: 0.13, SE: 0.02, *p <* 0.0001). Likewise, the Order of Target-Distractor trials caused a significant delay in RTs relative to Distractor-Target trials (*β*: 0.06, SE: 0.02, *p <* 0.01). The full model output is also available in Appendix A (Table A2). Figure 3 illustrates the effect of Order and Plausibility by Group. To examine whether the cost caused by the main effects was cumulative, we conducted a follow-up comparison across the four conditions with FDR-corrected pairwise t-tests, separately for each language group. For both groups, the results revealed significant differences between plausible TD and both implausible DT trials (all *p* < 0.001) and implausible DT (all *p* < 0.05). Differences between implausible DT and plausible DT were significant in non-native speakers (*p* < 0.05) and marginally significant for monolinguals (*p* = 0.05).

### 3.3. Discussion of Behavioral Results

Implausible DT trials (e.g., *shoot – EAT – breakfast*) represent the baseline level, both conceptually and in the statistical analyses. The implausibility of the distractor verb–noun combination means that the level of conflict between distractor and target verbs is kept to a minimum. Likewise, the fact that the target verb follows the distractor and appears just before the noun, should produce no cost. If anything, it should produce facilitation in the form of “collocational priming” [22,70,71], given that the target verb (*eat*) is followed by a predictable noun (*breakfast*), in consonance with previously experienced multiword units. The current paradigm reveals the expected behavioral costs from two independent factors. The results showed that the simple effect of Order (as seen in the implausible distractor with TD order condition) caused a significant slowdown in RTs. Distractor Plausibility had a similar effect, as seen in plausible DT trials, in which the cost is generated by a plausible distractor, but with no additional cost on account of the baseline DT order. The reported pairwise comparison confirmed that differences in signficance increased as effects accumulate. Specifically, plausible TD trials, which induced a cost from both factors, show that the cost is in fact doubled, suggesting a cumulative effect of selecting between two plausible candidates when the distractor also precedes the noun.

However, the results of the accuracy analysis are only partly congruent with the RT data, and provide additional insight into the nature of the costs of selection across groups. Accuracy in plausible distractor trials largely mirrored the effects of Order and Plausibility across both groups. On the other hand, the interactions of these two factors with Group revealed a different picture when implausible distractors had to be rejected. Native speakers’ accuracy was lower when an implausible distractor immediately preceded the noun, indicating that the processing of an implausible V-N sequence (e.g., *shoot breakfast*) incurred a cost. On the contrary, this resulted in an advantage for L2 speakers, suggesting that processing was not linear, unlike that of natives. That is, the advantage in rejecting implausible phrases seems to indicate that Verb and Noun were not processed as a unit, but rather that bilingual speakers might have focused on the plausibility of each candidate. This interpretation would be in line with theories that propose that non-native speakers’ processing relies more on plausibility and less on other linguistic cues than native speakers’ (e.g., the Shallow Structure Hypothesis [72]). We return to this idea and discuss it in more depth in the general discussion.

## 4. ERP Analysis

We present the results of the ERP analysis in two subsections, one for each of the language groups. Given that there is no prior literature that has identified the ERP components of conflict detection and resolution during multiword lexical selection, we take an exploratory approach to define the time window for analysis that is relevant for each group. To do this, we first relied on mass univariate analysis to identify the time windows of interest for the analysis, and then conducted repeated measures ANOVAs on those time windows. This approach involves conducting a statistical test at each time point and electrode of interest that is then corrected to control the Type I error or false discovery rate, offering a more objective and systematic way to determine time windows and regions of interest (see Section 2.4 for details of implementation in this analysis).

Additionally, because there was no apriori justification to assume identical ERP components in native and non-native speakers, we conducted separate analyses for each group. This decision is grounded on a considerable number of processing studies showing that processing in L1 and L2 speakers may elicit different ERP components and/or in different time windows, even when conducting the same task with identical stimuli [73,74,75,76].

### 4.1. ERP Analysis of Native English Speakers

**Figure 4:**
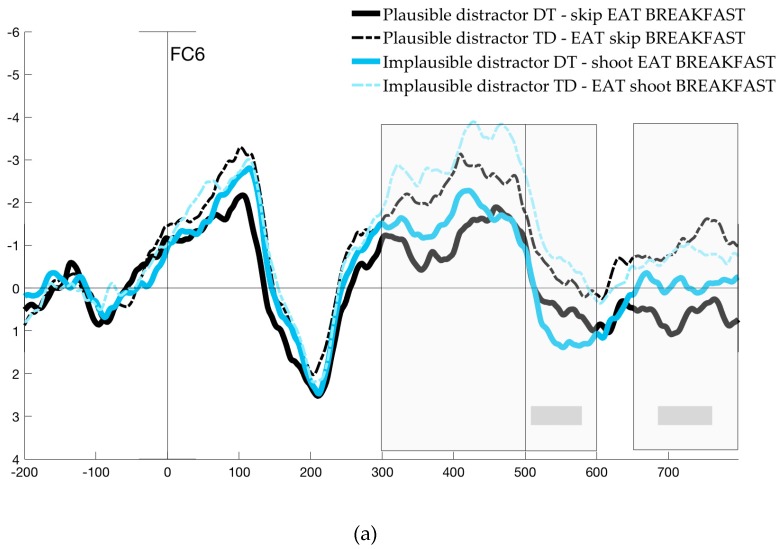

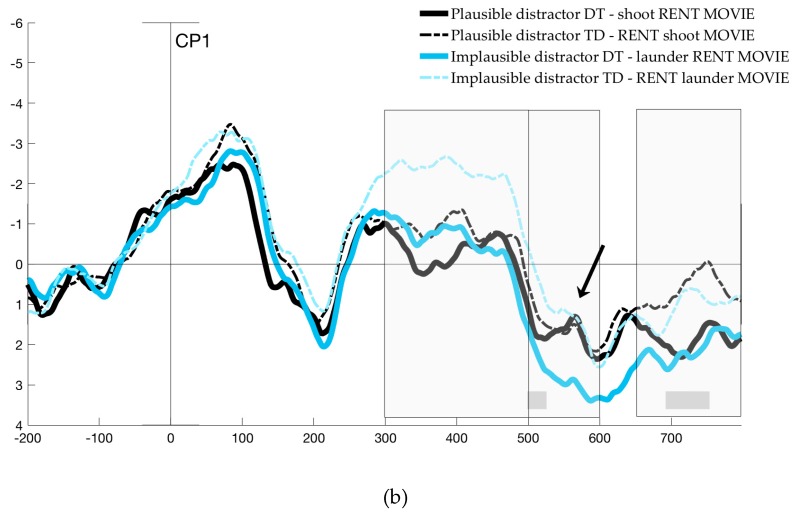
ERP waveforms at (**a**) FC6 (right frontocentral) and (**b**) CP1 (left centroparietal). The legend shows examples for each condition, with the target verb capitalized. Grey bars underneath show the time points of significant effects in running *t*-tests within the pre-defined 500–800 ms time window. Boxes show the three time windows analyzed. The arrow in (b) indicates the N_inc_ effect. Negativity is plotted up.

**Figure 5:**
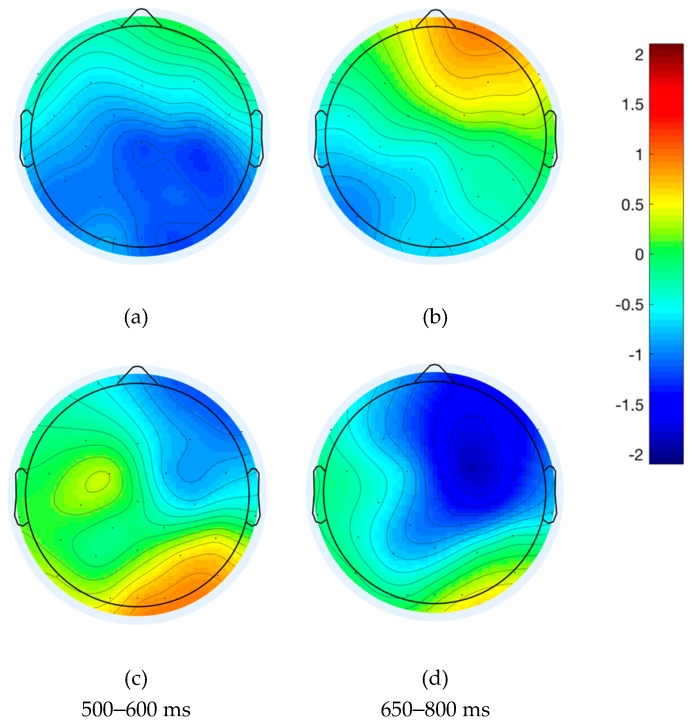
Scalp topographies of the difference waves showing the effects of (**a**,**b**) Distractor Plausibility (Plausible-Implausible) and (**c**,**d**) Order (TD – DT in plausible trials) in the 500–600 ms and 650–800 ms time windows, respectively.

The ERP analysis focused on three different time windows: the 300–500 ms time window (the N400 component) and another two time windows (500–600 ms and 650–800 ms) revealed by significant differences in the exploratory mass univariate analysis. This analysis revealed significant differences modulated by Order, mostly distributed over right frontal and left posterior electrodes (F4, F8, FC6, FC2, CZ, C4, CP1, CP5, C4, CP2, PZ, P3). Significant differences between waves aroused between 500 and 600 ms after presentation of the noun, as well as at a later time interval around 730 ms. Significant time windows are illustrated by grey bars in Figure 4a,b for two representative electrodes (plots for six additional representative electrodes are provided in Figures S1–S6 of the supplementary materials). Because mass univariate analysis is likely to underestimate the true boundaries and differences between waveforms due to stringent correction procedures [60,77], we conducted additional ANOVAs in the time windows around these peaks, between 500 and 600 ms and 650 and 800 ms. The results for these three time windows analyzed are presented below, and scalp maps are presented in Figure 5.

#### 4.1.1. N400 Component (300–500 ms)

The midline ANOVA revealed a main effect of Order (*F*(1, 22) = 11.57, *p <* 0.05), showing a significant difference in the N400 of DT and TD trials. There was also a main effect of Frontality (*F*(1.07, 23.58) = 32.17), but no interactions and no effect of Plausibility. The lateralized ANOVA also revealed main effects of Order (*F*(1, 22) = 12.54), and Frontality (*F*(1.07, 23.5) = 50.78). Additionally, there were three-way interactions between Plausibility, Hemisphere and Frontality (*F*(2, 44) = 4.79), and Order, Hemisphere and Frontality, (*F*(2, 44) = 9.82), showing that the effect of both Plausibility and Order differed across scalp regions. In post-hoc pairwise comparisons, we examined the simultaneous effect of Plausibility and Order for each scalp region. Across all regions, there was a significant effect of Order for trials with implausible distractors; that is, TD trials (e.g., *EAT – shoot – breakfast*) elicited a greater negativity than DT trials (*shoot – EAT – breakfast*). But when both verbs were plausible (e.g., *EAT – skip – breakfast*), differences elicited by Order were only significant in right frontal electrodes, with TD eliciting a greater N400 relative to DT (*t*(91) = −4.48, *p <* 0.001).

Additionally, in trials in which distractors preceded the noun (TD), the effect of Plausibility (e.g., *EAT – skip – breakfast* vs. *EAT – shoot – breakfast*) was most significant over left posterior electrodes (*t*(91) = 4.87, *p <* 0.0001), and only marginally significant in left frontal electrodes (*t*(91) = 2.14, *p* = 0.07). In Distractor-Target trials, Plausibility was not significant over any of the regions. Overall, differences were most prominent in the right frontal region, where comparisons were significant across all conditions except for implausible and plausible Distractor-Target trials. In left and right posterior electrodes, differences reached significance between implausible TD trials and each of the other conditions only. Figure 4a,b show two representative electrodes.

#### 4.1.2. 500–600 ms Time Window

The analysis of the midline electrodes showed main effects of Order (*F*(1, 22) = 5.24, *p <* 0.05) and Frontality (*F*(1.09, 24.02) = 25.21, *p <* 0.0001). Similarly, the lateralized ANOVAs also revealed main effects of Order (*F*(1, 22) = 7.22, *p* < 0.05) and Frontality (*F*(2,44) = 31.67, *p <* 0.0001), as well as three-way interactions of Plausibility, Hemisphere and Frontality (*F* (2, 44) = 5.5, *p <* 0.01), and Order, Hemisphere and Frontality (*F* (2,44) = 16.08, *p* < 0.0001). Post-hoc comparisons revealed significant differences between implausible Distractor-Target trials (*shoot – EAT – breakfast*) and all other trial types in left frontal, and left and right posterior electrodes; differences were most highly significant in the left posterior region (*p* < 0.0001). This confirmed that the negative peak between 500 and 600 ms was present in all conditions except for implausible Distractor-Target trials. Over right frontal electrodes, plausible Distractor-Target (*skip – EAT – breakfast*) was also different from both implausible TD (*EAT – shoot – breakfast*; *t*(91) = 4.84, *p <* 0.0001) and plausible TD (*EAT – skip – breakfast*; *t*(91) = −2.61, *p <* 0.05).

#### 4.1.3. 650–800 ms Time Window

As in the other two time windows analyzed, main effects of Order (*F*(1, 22) = 7.54, *p <* 0.05) and Frontality (*F*(0.9, 19.84) =14.26, *p* < 0.001) were revealed by the midline electrode analysis. In the lateralized ANOVA, the same effects were found for Order (*F*(1, 22) = 7.28, *p <* 0.05) and Frontality (*F*(1.1, 23.28) = 17.5, *p <* 0.0001), and again, three-way interactions between Plausibility, Hemisphere and Frontality (*F*(2, 44) = 5.28, *p <* 0.01) and Order, Hemisphere and Frontality (*F*(2, 44) = 25.39, *p <* 0.0001). Pairwise *t*-tests revealed no differences in any of the comparisons in left frontal and right posterior regions. In right frontal and left posterior electrodes, differences were significant for each comparison, with the exception of implausible vs. plausible Target-Distractor trials (*EAT – shoot – breakfast* vs. *EAT – skip – breakfast;* right frontal, *t*(91) = −0.79, *p* = 0.44; left posterior, *t*(91) = −0.47, *p* = 0.64).

#### 4.1.4. Correlation between Neurophysiological and Behavioral Data in Native Speakers

Finally, exploratory tests were performed to investigate the potential correlation between ERP amplitude difference waves and individual behavioral data. To investigate the correlation between the effect of Plausibility in ERP and behavioral data, first the individual N_inc_ amplitudes were calculated by subtracting the average amplitudes of Plausible DT-Implausible DT in the 500–600 ms time window. The same subtractions were performed on the individual averages of RT and accuracy data. The results revealed a marginally significant correlation between the N_inc_ and the difference in RTs of Plausible DT-Implausible DT (*r =* −0.38, *p* = 0.08). However, no correlation was found between individual N_inc_ measures and accuracy.

To investigate the effect of Order, the ERP difference wave was calculated for TD-DT comparison within the 650–800 ms time window for both the Plausible and Implausible distractor conditions over left posterior and right frontal electrodes. The same subtractions were performed on individual RT and accuracy averages. In contrast with the previous comparison, this time the results revealed a significant correlation between the ERPs and the proportions of accurate responses between Plausible TD-Plausible DT in both left posterior (*r* = −0.48, *p* < 0.05) and right frontal (*r =* −0.54, *p* < 0.01). However, no correlation was found between individual ERP measures from 650–800 ms and RTs. For Implausible trials, correlations were non-significant. We further interpret these results in the Discussion.

### 4.2. Discussion of ERP Results in Native Speakers

In the analysis of the monolingual ERP data, we aimed to investigate the neural correlates of conflict detection and resolution in native speakers of English, in a task in which they had to select the correct verb out of two options presented to form a familiar multiword unit. Differences across conditions were examined by comparing trials in which the distractor verb was plausible or implausible, and based on order (target verb presented last or distractor presented last). In the ERP analysis we further disentangle different types of cost by examining three time windows locked to the presentation of the noun. The analysis of the N400 window allowed us to examine differences in the processing of the noun resulting from priming, or lack thereof, from verbs that preceded it. The results showed the expected modulations within the prototypical time window and parietal scalp distribution of the N400. Left and right posterior electrodes reflected significant differences between conditions in which the noun was primed by the verb relative to the one condition in which the verb was an implausible distractor. That is, in Distractor-Target trials, in which the noun was immediately preceded by the target verb (both *skip – EAT – breakfast* and *shoot –EAT – breakfast*), the N400 was attenuated, regardless of distractor plausibility. Critically, the same was true for trials in which the noun was preceded by a distractor that was plausible (*EAT – skip – breakfast*). However, as illustrated in Figure 4b, these conditions were all different from cases in which there was no priming possible, namely, in Target-Distractor trials that presented an implausible verb before the noun (*EAT –shoot – breakfast*). Interestingly, right frontal electrodes (as illustrated in Figure 4a) revealed a gradient effect modulated by both Plausibility and Order, such that nouns primed by both the target and a plausible distractor show the most attenuated N400.

The following two time windows based on the mass univariate analysis (500–600 ms; 650–800 ms) allowed us to address the critical predictions regarding the selection of the verb in native English speakers. In the 500–600 ms time window, a negative-going peak was elicited in the two conditions in which conflict was caused by a competing plausible verb (black lines in Figure 4). The scalp topography of this effect (Figure 5a) was also consistent with the left centro-parietal distribution of the N_inc_ reported in previous studies [24,25,26,27,28,29,34,35,36,37,41,42]. The result of the N_inc_ elicited by plausibility-induced conflict is consistent with our second prediction regarding conflict due to interference in selection. Namely, we expected that the N_inc_ would be elicited by trials in which distractors are plausible candidates for selection (e.g., *skip* in *skip – EAT – breakfast*), at some point between the processing of the noun and before responses were made.

Additionally, the same negative deflection appeared to be elicited by the Order effect alone within the 500–600 ms time window in trials with an implausible distractor in which the non-target verb was presented last (i.e., implausible TD, *EAT – shoot – breakfast*). This would suggest a cost of Order; however, it must be noted that the comparison between plausible TD and plausible DT for that same time window produced no effect of Order. Because the implausible TD trials are the only ones in which a greater negativity was elicited during the N400 time window, it seems possible that this sustained negativity may be a carry-over effect. In fact, this would also explain why a trending significant correlation was found between the Plausibility difference wave in this time window and RTs, but not for Order. Due to these observations, we note that the plausibility effect seems robust, but the sustained negativity in implausible TD trials after 500 ms should be taken with caution.

Differences in the later 650–800 ms time window showed that there was also a main effect of Order, for both plausible and implausible trials. This effect was significant in left posterior electrodes and largest in right frontal areas, with a significant negativity found in distractor-last trials (TD) relative to target-last (DT). This Order effect is in line with the assumption that the Target-Distractor trials pose an increased demand for controlled selection, given that participants must discard the most recently (and therefore strongly) activated candidate. However, it is only partly congruent with the LPC reported in previous studies. While it matches the inverted negativity for conflict conditions in frontal electrodes, the elicited wave is also more negative for TD trials; that is, the polarity of the effect does not differ in left posterior electrodes.

Given these predictions and the topography of this effect, we interpret it in connection with the literature on memory and retrieval. The functionality and scalp distribution of this negativity seem to be congruent with the left parietal effect and right-frontal effects (RFE) of memory retrieval studies conducted using the old/new paradigms. The left parietal old/new effect elicits a more positive wave for correctly retrieved items, and a more negative wave in correct rejections ([78,79,80]; however, other studies have reported mixed results, with similar amplitudes for inaccurate recollections [81,82]). Although the functionality of these components is still debated, it has been suggested that it is associated with memory retrieval. Relevant to the hypotheses tested here, some studies have found a correlation between the amplitude of the effect (referred to as LPC in some studies, e.g., [83]) and response accuracy in line with our own data.

Importantly, it has been proposed that this component reflects decisional factors during memory retrieval, including accuracy and possibly confidence in the response. The similarities with the current paradigm are quite apparent, given that distractor-last trials require the evaluation and monitoring of the accessed representation (distractor – noun) and its rejection based on the phrases presented earlier during the study.

However, as noted, the effect was present most prominently in right frontal electrodes in addition to left posterior electrodes. The right-frontal old/new effect (RFE) has been associated with “post-retrieval” monitoring processes (e.g., [84,85,86]). The RFE is associated with monitoring of retrieved episodic information while a judgement is made (i.e., post-retrieval monitoring, [83,87,88]). Similarly, here distractor-last trials required the evaluation and monitoring of the accessed representation (distractor – noun), and their rejection based on the phrases presented earlier during the study. Crucially, the results of the analysis, showing that the difference waves between 650 and 800 ms correlated with accuracy, support the interpretation of this negativity as reflecting monitoring processes during selection.

A clarification about the labels of components seems necessary here. As Finningan and colleagues point out, the terminology is inconsistent across studies within the ERP literature on retrieval, with the same component sometimes being referred to as LPC, P3, P300, or P600. More confusingly, the same label (LPC) refers to a positivity in *known* items in these studies for which retrieval is successful, and is believed to index a matching representation, while in the LPC in the Stroop literature, the positivity is associated with a *cost* in selection for incongruent (conflict) trials. In other words, while the literature on retrieval emphasizes the polarity of trials with successful recollection (positivity), the conflict literature describes the polarity of conflict trials (negativity).

Given that the peak of the effect was present in right frontal electrodes, and in order to follow previously employed terminology, we will refer here to this right frontal late negativity as the right frontal effect (RFE), while emphasizing the association of a negativity with a cost in retrieval during selection. 

All in all, the effects identified across the three time windows allow us to differentiate the aggregated effects reflected in the behavioral data, providing a finer-grained analysis of the qualitative costs incurred during lexical selection. To summarize, consistent with RT results, the cost of processing the noun in implausible TD trials, as well as the cost associated with distractor-last trials, accounts for the significantly slower RTs relative to implausible DT. Plausible trials elicited a conflict-detection component (the N_inc_) within the 500–600 ms time window. Finally, plausible TD trials showed an Order effect in the negativity from 650–800 ms, maximal over right frontal electrodes, as well as the longest RTs in the behavioral results.

### 4.3. ERP Analysis of Spanish–English Bilinguals

The behavioral results reported above showed that averaged RTs started shortly after 950 ms in bilinguals; that is, there was a delay of approximately 150 ms relative to native speakers. Therefore, the mass univariate analysis was adjusted accordingly to examine the time window from 500 to 950 ms. Because extending the time window of the exploratory analysis involves an increased loss of statistical power due to more comparisons being made, the analysis was restricted to the electrodes of areas of interest; these were the left centroparietal and right frontal areas of the native English speakers, where the effects were expected.

**Figure 6:**
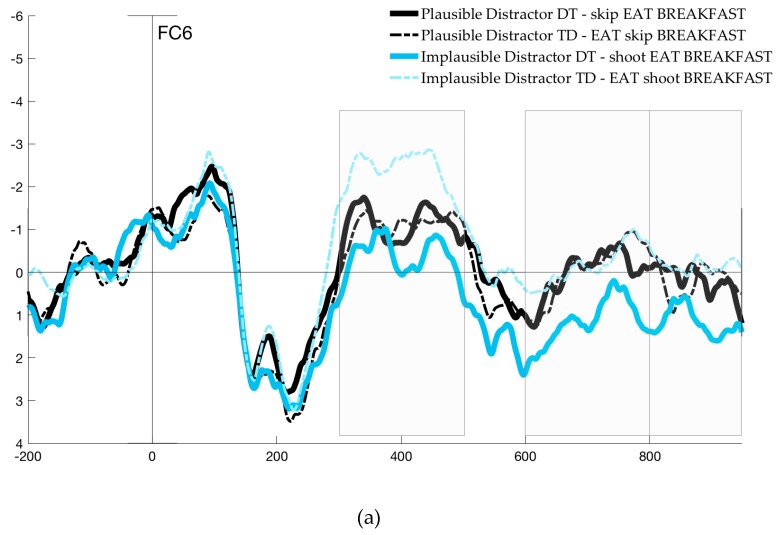

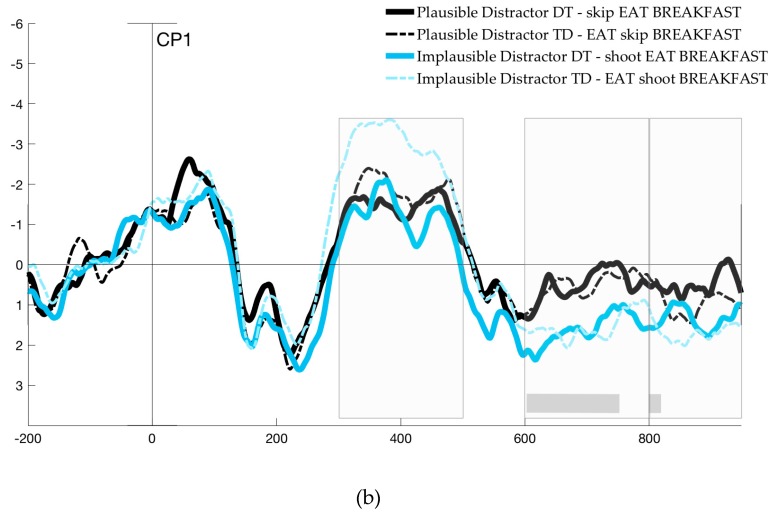
ERP waveforms for non-native speakers at (**a**) FC6 (right frontocentral) and (**b**) CP1 (left centroparietal). The legend shows examples for each condition, with the target verb capitalized. Grey bars underneath show the time points of significant effects in running *t*-tests within the pre-defined 500–950 ms time window. Boxes show the three time windows analyzed. The arrow in (b) indicates the N_inc_ effect. Negativity is plotted up.

For the non-native speakers, running *t*-tests revealed differences modulated by Plausibility over left posterior electrodes (CP1, CP5, P3, P7). A sustained effect was found from 600 to 800 ms, with more irregular intervals of significance across the same electrodes between 800 and 900 ms (significant intervals are illustrated for CP1 by gray bars in Figure 6; Figures S7–S13 of the supplementary materials show the differences from running *t*-tests for each significant electrode). The fact that the significant effect appeared later is congruent with the delay observed in RTs. Unlike in the native speaker group, no other differences were revealed. Nonetheless, based on the late right frontal negativity (RFN) found in the native group during the final 150 ms preceding the beginning of RTs, and for comparability between native and non-native speakers, it was deemed worth investigating this final time window. The ERP analysis therefore considered three time windows: 300–500 (N400), 600–800 ms and 800–950 ms.

#### 4.3.1. N400 Component (300–500 ms)

The midline ANOVA revealed main effects of Order (*F*(1, 20) = 6.56, *p* < 0.05) and Frontality (*F*(2, 42) = 7.72, *p* < 0.01), and an interaction of Plausibility x Order (*F*(1, 20) = 5.0, *p* < 0.05). Similarly, the lateralized ANOVA also showed main effects of Order (*F*(1, 20) = 7.78, *p* < 0.05) and Frontality (*F*(2, 42) = 11.71, *p* < 0.0001), as well as interactions of Plausibility and Order (*F*(1, 20) = 6.32 *p* < 0.05) and Order x Hemisphere x Frontality (*F*(1.05, 20.91) = 4.1, *p* < 0.05). Pairwise comparisons showed that, crucially, implausible TD trials (*EAT – shoot – breakfast*) were different from all other conditions over left and right posterior electrodes (all *p* < 0.0001), with no other significant differences. In frontal electrodes, all comparisons showed significant differences except for plausible DT vs. plausible TD.

#### 4.3.2. 600–800 ms Time Window

**Figure 7:**
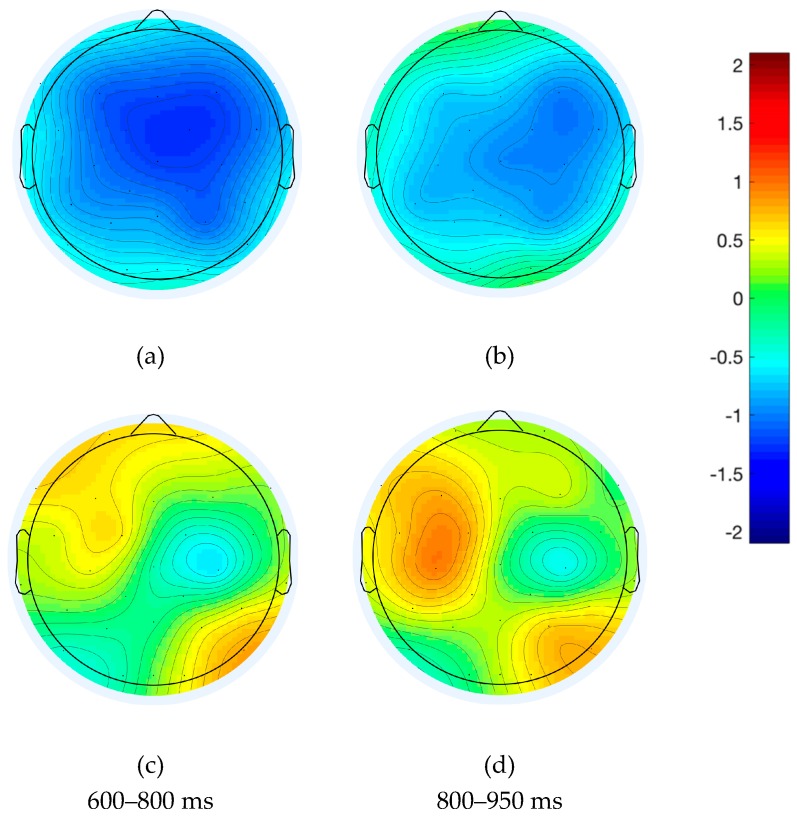
Scalp topographies of the difference waves in non-native speakers, showing the effects of (**a**,**b**) Distractor Plausibility (Plausible–Implausible) and (**c**,**d**) Order (TD–DT in plausible trials) in the 600–800 ms and 800–950 ms time windows, respectively.

The results of the midline ANOVA revealed main effects of Plausibility (*F*(1, 20) = 5.12 *p* < 0.05) and Frontality (*F*(2, 40) = 4.6, *p* < 0.05). In the lateralized analysis, there were main effects of Plausibility (*F*(1, 20) = 5.09, *p* < 0.05) and Frontality (*F*(2, 40) = 5.8, *p* < 0.01), and interactions between Order x Hemisphere (*F*(1, 20) = 4.57, *p* < 0.05) and Hemisphere x Frontality (*F*(2, 40) = 6.9, *p* < 0.01). Three-way interactions emerged between Plausibility x Hemisphere x Frontality (*F*(2, 40) = 3.41, *p* < 0.05), and Order x Hemisphere x Frontality (*F*(2, 40) = 71.91, *p* < 0.0001). The three-way interactions suggest that the main and effects of Order and Plausibility differed across scalp regions. Pairwise comparisons confirmed that, in left and right posterior electrodes, differences were significant only between plausible and implausible trials, with no effect of Order. This was also the case in left frontal electrodes, although comparisons for plausible TD were marginally significant with plausible DT (*t*(83) = 1.99, *p* = 0.07) and not significant with implausible DT (*t*(83) = −1.57, *p* = 0.11). Over right frontal electrodes, implausible DT (*shoot – EAT – breakfast*) was different from all other conditions (*p* < 0.01), with no other differences. Difference waves in Figure 7a,b show the scalp distribution of the effects, with a widely distributed effect of Plausibility (7a).

#### 4.3.3. 800–950 ms Time Window

The midline ANOVA only showed a significant effect of Frontality (*F*(2, 40) = 11.36, *p* < 0.0001). In the lateralized analysis, significant main effects were found for Frontality (*F*(2, 40) = 9.0, *p* < 0.001) and Hemisphere (*F*(2, 40) = 5.77, *p* < 0.05). There were significant interactions of Order x Hemisphere (*F*(1, 20) = 9.63, *p <* 0.01), Hemisphere x Frontality (*F*(2, 40) = 6.88, *p* < 0.01), Order x Hemisphere x Frontality (*F*(2, 40) = 54.31, *p* < 0.0001) and Plausibility x Order x Hemisphere x Frontality (*F*(2, 40) = 4.69, *p* < 0.05). Pairwise comparisons showed the same effects as in the previous time window for left posterior electrodes, with significant differences only between plausible and implausible conditions, and no effect of Order. Over right frontal electrodes, once again implausible DT (*shoot – EAT – breakfast*) was less negative than all other conditions (*p* < 0.001).

### 4.4. Correlation between Neurophysiological and Behavioral Data

Based on the results of the experiment with native speakers, we performed one-tailed correlations between individual ERP amplitude differences waves and the behavioral results. Differences between plausible and implausible DT trials within the 600–800 ms and 800–950 ms time windows were not correlated with accuracy nor RTs. Correlations between the individual familiarity ratings for multiword units and amplitude difference waves were also non-significant.

### 4.5. Discussion of ERP Results in Non-Native Speakers

We used the same materials and paradigm as with native speakers to investigate the ERP components associated with conflict detection and resolution in late Spanish–English bilinguals. The results revealed that the plausibility manipulation produced similar effects in non-native speakers of English, but there were also some notable differences. The behavioral results showed similar patterns to those found in native speakers. However, in both the analysis of accuracy and in RTs, only a main effect of Plausibility emerged; there was no significant main effect of Order. In the analysis of accuracy, the interaction of Order x Plausibility was significant, showing that non-native speakers made more errors when distractors were plausible and were presented last (*EAT – skip - breakfast*). Additionally, unlike in native speakers, the measure of English verbal fluency (which accounted for individual language ability in English) was a significant predictor of overall performance in the task, In the ERP analysis, the N400 component showed the expected cost in processing the noun in implausible TD trials (*EAT – shoot – breakfast*). Interestingly, a significant difference emerged in the right posterior region between plausible TD and implausible DT, but not between plausible TD and DT. This suggests that processing the noun did not seem to significantly differ between trials in which it was immediately preceded by a plausible distractor and trials in which it was preceded by the target.

The analysis of the 600–800 ms time window showed a widely distributed effect of Plausibility in the comparison between plausible and implausible target-last trials. As shown in Figure 7a,b, the effect was widely distributed; however, there was no effect of Order, as shown in Figure 7c,d. Over right frontal electrodes, the baseline condition significantly differed from all others, showing a generalized cost of both Order and Plausibility. The absence of an Order effect in the comparison between plausible TD and DT, with both showing a negativity relative to the baseline, suggests that rather than no cost in distractor-last trials, there was simply no advantage in target-last trials. That is, the results suggest that lexical selection in trials with plausible distractors produced a generalized cost for non-native speakers. The absence of a facilitation in DT trials appears to be caused by an increased need for monitoring during retrieval in bilinguals.

Additionally, a more generalized cost was widespread across scalp locations (Figure 7a,b). More importantly, unlike in native speakers, no two components could be separated, although differences in the effect over right frontal became more highly significant between 800 and 950 ms. The impossibility to temporally separate different components may be due to a greater degree of variability among non-native speakers, i.e., in their proficiency or other individual differences regarding the L2. Even though we restricted our sample to late non-simultaneous Spanish–English bilinguals immersed in their L2, processing in the non-native language is known to be more variable and other aspects of their experience and learning might have introduced additional variables. However, an alternative explanation is possible. We suggest that, in non-native speakers, processing may be more costly in terms of retrieval and monitoring, demanding engagement of more cognitive resources. Therefore, there may be increased overlap in the timing of conflict detection and monitoring in bilinguals. We elaborate on this idea in the General Discussion.

In summary, the ERPs of bilingual participants showed a main effect of Plausibility, in line with the behavioral data. The convergence of behavioral and ERP data suggests qualitative differences in the bilingual group relative to native speakers, in that Plausibility but not Order seemed to pose a cost in selection. We turn to this question in connection with similar prior evidence in bilinguals in the last section.

## 5. General Discussion

The present study examined the cognitive mechanisms involved in conflict resolution in native and non-native speakers during lexical selection in a context where selection takes place in units larger than a single word (e.g., *eat breakfast*). To do so, we employed a paradigm with sequential presentation of two verbs and a noun (e.g., *EAT – skip – breakfast*). The materials were manipulated so that distractors would either be plausible in English (as in the previous example) or implausible (*EAT – shoot – breakfast*). Importantly, in the case of non-native speakers (i.e., late Spanish–English bilinguals), the plausible distractor + noun combinations formed multiword units that were incongruent with bilinguals’ native language (*skip breakfast* is “saltarse el desayuno”, ‘to jump the breakfast’). The order of presentation of the distractor verb and the target verb was counterbalanced (*EAT – skip* or *skip – EAT*). This 2 x 2 design allowed us to examine (a) how the verb that was presented most recently affected the processing of the noun (i.e., priming), during the N400 window; (b) the conflict-related ERP components that are elicited in high conflict contexts (both verbs are plausible) relative to low conflict contexts (implausible distractor); (c) the ERP correlates of conflict-resolution and monitoring prior to response selection.

The results of the experiment showed that the processing of the noun was facilitated by priming from the verb in the native speaker data when the target immediately preceded the noun (*eat breakfast*), but also when a matched plausible distractor primed the noun (*skip breakfast*). In both datasets, ERPs time-locked to the presentation of the noun showed an attenuated N400. The same pattern was found in the bilingual speaker dataset. Interestingly, neither the analysis of the native nor non-native speakers’ data showed significant differences between trials in which the noun was primed by the target verb (e.g., *EAT*) or by the plausible distractor (*skip*). This is remarkable because target verbs + nouns formed multiword units congruent across the L1–L2, while plausible distractors + nouns formed L1–L2 incongruent multiword units.

Taking direction from the psycholinguistic literature on conflict detection, we expected that the N_inc_, a conflict-related component described in the ERP studies using the Stroop paradigm, should emerge in the time period after the processing of the noun and before a response was made. Results for native English speakers showed a negativity (500–600 ms), maximal in left centroparietal electrodes. This effect was congruent with the functionality and scalp distribution of previous studies that identified the N_inc_ using the Stroop paradigm. The negativity was modulated by Plausibility, showing that interference from a valid candidate elicited the N_inc_ component; it was also present when conflict arose from the need to discard an implausible target that was presented immediately before the noun. The fact that the N_inc_ would be elicited for different types of conflict is in line with the evidence that conflict-detection is a domain-general mechanism, elicited by linguistic and non-linguistic conflict (i.e., visual conflict in direction, as in the Flanker task; semantic conflict mediated by modality, as in Stroop; [13]). Additionally, a marginally significant correlation between ERP difference waves and RTs suggested an association between conflict detection and the delay of responses.

In addition, monolinguals showed a later component before responses were made. The functionality and scalp distribution of this negativity were congruent with the right-frontal old/new effect (RFE), believed to reflect general “post-retrieval” monitoring processes (e.g., [84,85,87,88]). The effect is associated with monitoring of retrieved episodic information while a judgement is made (i.e., post-retrieval monitoring, [88]). The results of the correlations showing that the difference waves between 650 and 800 ms correlated with accuracy support both the interpretation of this negativity as the RFE as well as the proposal that this component reflects post-retrieval monitoring that supports accurate selection. The component was observed to be modulated in native speakers by Order affecting the selection of the target when verbs competed for selection. Importantly, the analysis showed that the amplitude of the RFE was significantly correlated with the proportion of accurate responses. This provides compelling evidence that this potential is associated with monitoring during goal-oriented selection of the target.

Results for bilingual speakers revealed important similarities but also some different patterns. First, similarly to the analysis of the native speaker data, the bilingual data showed that plausible verbs primed the noun within the 300–500 ms time window, and this was the case for both target verbs (*eat breakfast*) or plausible distractors (*skip breakfast*), despite the cross-language differences for non-native speakers. Second, preceding the selection of the target verb, ERPs also showed significant differences modulated by Plausibility. Similarly to the native speaker data, the effect was maximal over left centroparietal and right frontal electrodes. However, there were also some notable differences regarding the timing of selection-related components. While in native speakers an N_inc_ was followed by a right frontal effect (RFE), indicating a cost in post-lexical retrieval, in non-native speakers, the timing of both effects was indistinguishable. Maxima were found over left centroparietal and right frontal electrodes in both groups, but in non-native speakers the effect manifested in the form of a more sustained and widespread negativity. This conflict-related negativity was more prolonged in time (600–800 ms), appeared to amalgamate both the N_inc_ and RFE, and became more prominent in right frontal electrodes shortly before responses were made (800–950 ms).

We suggest that the findings of the non-native speakers are in line with our the current understanding of cross-language activation in bilinguals. On the one hand, it is not unexpected that non-native speakers should experience increased demands for monitoring and selection. The need for sustained or prolonged monitoring would be justified by the fact that bilinguals activate more information than monolinguals, including activation from multiword units in the L1 [19,89]. While we refrain from making any specific claims regarding the brain areas engaged in the two populations tested, there is good evidence from neuroimaging studies that using the weaker language results in greater and more widespread brain activation in bilinguals (for a recent review, see [90]). For instance, a “prefrontal effect” is associated with usage of the less dominant language [91]. These cortical regions are not involved in conflict detection but in the process of response selection and suppression [92]. Additionally, increased activation in the left inferior parietal lobule is associated with biasing lexical selection towards the language in use [93]. In light of this evidence, it is not surprising that bilinguals would show a more generalized cost, manifested in the widespread effect revealed in our data. Also, because the effect was more extended in time, this might suggest a need to maintain and re-evaluate competitors in memory. Thus, the overlapping of monitoring and selection components may reflect a processing cost due to less entrenched representations of multiword units in the L2 and uncertainty in the correct lexical choice. This would be in line with some accounts suggesting that the RFE may indicate a greater number of computations required in response selection [86,94]. The current data do not allow us to determine the loci of the costs, and these interpretations will need to be tested in future research.

Further, beyond the timing of the processes, the significance of the effects also showed both similarities and differences in behavioral and ERP data. Behaviorally, there was a main effect of Plausibility in the RTs of both native and non-native speakers, as well as a main effect of Order. However, the patterns found in the accuracy data revealed that the effect of Order was robust only in the native speakers, who consistently showed a cost in distractor-last trials. In contrast, non-native speakers did not show a reliable effect of Order. In fact, when implausible distractors were presented last, bilinguals showed an *advantage* in selection. These patterns in the accuracy data are indicative of differences in the processing or selection strategies across groups, with Order having a more consistent effect in native speakers, while bilingual speakers are consistently affected by Plausibility. While the effect of Group was tested directly only in the behavioral analysis, we note that the by-group ERP results were consistent with these patterns. In native speakers, the conflict-detection component over left centro-parietal electrodes showed a negative peak for trials with plausible distractors as well as for target-last trials; in non-native speakers, left centroparietal electrodes showed a significant effect of Plausibility but not Order, in line with the suggestion that conflict-detection was not modulated by Order in bilinguals.

Why would plausibility, rather than order, have a greater effect in non-native speakers than in native speakers? The answer may lie in known differences between processing in the L1 and the L2 in general and, more specifically, in the processing of multiword units by adults. Current theories of sentence processing, such as the Shallow Structure Hypothesis (SSH, [95,96,97]), posit that non-native speakers often achieve native-like performance in online processing tasks, but do so by relying on non-grammatical (e.g., lexical) cues to a greater extent than on syntactic and morphosyntactic cues. To illustrate, evidence from online processing of syntactic subject/object ambiguities has shown that non-native speakers first rely on lexical and plausibility information, whereas reliance on plausibility information is typically delayed or absent in native speakers [98]. Therefore, the evidence suggests that when it comes to lexico-semantic interpretations, non-native speakers have greater sensitivity to plausibility.

An alternative, yet compatible, interpretation is based on the fact that monolinguals have entrenched knowledge of multiword units, and are sensitive to variations in form, i.e., expect to encounter them in a particular form. As mentioned in the introduction, previous research has shown faster processing in native speakers when a multiword unit is presented in its exact familiar form and order, e.g., “black and white” relative to “white and black.” Based on these findings, the order of the verbs before the noun may have played an important role in how monolinguals approached the task, potentially focusing on recognizing the expected verb–noun sequence. In addition to this, evidence has shown that L2 speakers are successful at learning individual words but they have difficulty in acquiring multiword units [99,100]. Indeed, it has been proposed that it is precisely non-native speakers’ greater focus on individual words and on decomposing multiword units that poses a constraint in acquiring L2 multiword units [23,101]. The discussed focus of non-native speakers on semantics, as well as the lesser reliance on structural cues, might have likely affected their approach to the task, with increased Plausibility-based costs, while relegating Order-based factors to a secondary stage. These factors may have together or independently contributed to the greater Order-based cost found in native speakers than in bilinguals. 

## 6. Conclusions

In the experiments presented, we investigated the brain potentials associated with conflict detection and resolution during multiword lexical selection. The novel paradigm employed allowed us to go beyond selection at the single word level to examine how, in forming everyday phrases, speakers detect and resolve conflict between the word choices available to them. When native and non-native English speakers were asked to select a familiarized target verb–noun sequence (*eat breakfast*), we observed that the context of selection modulated brain potentials. Specifically, contexts that resulted in conflict during selection due to more than one valid candidate induced negative-going potentials, as well as contexts in which the most recently processed verb had to be rejected (e.g., *eat – skip – breakfast;* target: *eat*). However, native English speakers seemed to experience a greater cost from Order of presentation (i.e., when the consecutively presented verb–noun sequence was not identified as the target). On the other hand, ERP components in late Spanish–English bilinguals were most significantly modulated by Plausibility. In other words, native speakers experienced a cost from not encountering the expected sequences of multiword units, while selection costs in non-native speakers were most affected by the plausibility of each individual combination. The bilingual finding is in agreement with current theories of processing that propose that, while native speakers rely on multiword units, non-native speakers processing is more focused on individual words [23,99,101].

## Figures and Tables

**Table 1:** Sample of experimental manipulation (*eat breakfast*).

Distractor Plausibility	Order	Verbs	Noun
Implausible	DT	shoot – EAT	breakfast
Implausible	TD	EAT – shoot	
Plausible	DT	skip – EAT	
Plausible	TD	EAT – skip	

**Table 2:** List of verb–noun phrases per condition.

Target List	Foil List	Plausible Distractors	Implausible Distractors
cancel [a] trip	schedule [a] time	schedule [a] trip	break [a] trip
eat breakfast	skip school	skip breakfast	shoot breakfast
finish [a] story	break [the] news	break [a] story	fail [a] story
promote peace	disturb [the] neighbors	disturb [the] peace	schedule peace
rent [a] movie	shoot photos	shoot [a] movie	launder [a] movie
save money	launder clothes	launder money	skip money
teach [a] class	fail [a] test	fail [a] class	cash [a] class
write [a] check	cash [a] prize	cash [a] check	disturb [a] check

**Table 3:** Summary of cognitive and proficiency measures.

	Native		Non-Native	
Measure	*M*	*SD*	*M*	*SD*
Age (in years)	23.09	3.6	32.17	5.97
L1 self-rated proficiency	9.83	0.36	9.84	0.36
L2 self-rated proficiency	3.37	2.01	8.58	0.63
MELICET (/100)	90.7	5.07	78.27	11.8
English Verbal Fluency	52.48	11.08	42.14	8.41
Flanker effect (ms)	48.87	23.65	47.85	23.64
Stroop effect (ms)	182.2	100.06	125.81	153.79
Familiarity L1–L2 congruent MWUs (/7)			6.45	0.65
Familiarity L1–L2 incongruent MWUs (/7)			5.51	1.23

**Table 4:** Results of accuracy in selection of the target verb by condition.

		Native		Non-Native	
Distractor Plausibility	Order	Mean Acc. %	SD	Mean Acc. %	SD
Implausible	DT	98.37	12.67	96.02	19.55
Implausible	TD	96.96	17.19	97.39	15.96
Plausible	DT	87.5	33.09	90.57	29.24
Plausible	TD	84.57	36.15	88.41	32.03

**Table 5:** Reaction times in selection of the target verb by condition.

		Native		Non-Native	
Plausibility	Order	Mean RT	SD	Mean RT	SD
Implausible	DT	815.82	348.02	977.31	363.71
Implausible	TD	838.69	337.65	994.7	349.05
Plausible	DT	858.94	399.93	1027.23	413.35
Plausible	TD	889.91	385.65	1043.07	394.62

## Supplementary Materials

The following are available online at https://www.mdpi.com/2076-3425/9/5/110/s1, Figures S1–S6: Additional electrodes (Fc2, F4, Cz, Pz, Cp5, P3) from native speakers, Figures S7–S13: Additional electrodes (Fc2, F4, Cz, Pz, Cp5, P3, P7) from non-native speakers.

## Appendix A

**Table A1:** Output of generalized linear regression accuracy analysis.

	Estimate	Std. Error	*z* Value	Pr(>|*z*|)	*p* Value
(Intercept)	4.47	0.34	13.03	0.00	<0.0001
Group Bilingual	−0.96	0.40	−2.40	0.02	<0.05
Plausible Distractor	−2.37	0.31	−7.66	0.00	<0.0001
Order TD	−0.65	0.32	−2.04	0.04	<0.05
Plausible Distractor: Order TD	0.39	0.35	1.14	0.26	0.26
Group Bilingual: Plausible Distractor	1.31	0.38	3.42	0.00	<0.01
Group Bilingual: Order TD	1.10	0.42	2.62	0.01	<0.01
Group Bilingual: Plausible Dist: Order TD	−1.14	0.47	−2.44	0.01	<0.05

**Table A2:** Output of mixed-effects regression RT analysis.

	Estimate	Std. Error	df	*t* Value	Pr(>|*t*|)	*p* Value
(Intercept)	−0.28	0.12	50.6	−2.28	0.03	<0.05
Group Bilingual	0.39	0.16	43.02	2.37	0.02	<0.05
Plausible Distractor	0.06	0.02	6224.44	3.07	0	<0.01
Order TD	0.13	0.02	6224.69	6.09	0	<0.0001

**Table A3:** Additional data for cognitive and proficiency measures.

	Native				Non-Native			
Measure	*M*	*SD*	skew	kurtosis	*M*	*SD*	skew	kurtosis
Age (in years)	23.09	3.6	2.37	5.16	32.17	5.97	0.62	−0.84
L1 self-rated proficiency	9.83	0.36	−1.6	0.79	9.84	0.36	−1.7	1.05
L2 self-rated proficiency	3.37	2.01	0.15	−1.24	8.58	0.63	0.28	−0.96
MELICET (/100)	90.7	5.07	−0.71	0.94	78.27	11.8	−0.38	−0.5
English verbal fluency	52.48	11.08	0.29	−0.3	42.14	8.41	0.47	−0.37
Flanker effect (ms)	48.87	23.65	−0.03	−0.24	47.85	23.64	0.28	−1.07
Stroop effect (ms)	182.2	100.06	1.08	0.65	125.81	153.79	0.53	0.49
Familiarity congruent MWUs (/7)					6.45	0.65	−1.52	1.48
Familiarity incongruent MWUs (/7)					5.51	1.23	−1.2	0.55

**Table A4:** Additional data for accuracy in selection of the target verb by condition.

		Native				Non-Native			
Distractor Plausibility	Order	Acc. %	SD	skew	kurtosis	Acc. %	SD	skew	kurtosis
Implausible	DT	98.37	12.67	−1.24	0.27	96.02	19.55	−2.14	4.7
Implausible	TD	96.96	17.19	−1.81	2.45	97.39	15.96	−0.78	−0.48
Plausible	DT	87.5	33.09	0.28	−1.28	90.57	29.24	−1.37	1.75
Plausible	TD	84.57	36.15	−0.61	−0.46	87.8	32.03	−0.33	−1.22
