# Supplementary figures and images for: The Neural Correlates of Conflict Detection and Resolution During Multiword Lexical Selection: Evidence from Bilinguals and Monolinguals
Source: Brain Sci. 2019 May 14;9(5):110. doi: 10.3390/brainsci9050110 (PMC6563143; doi:10.3390/brainsci9050110)

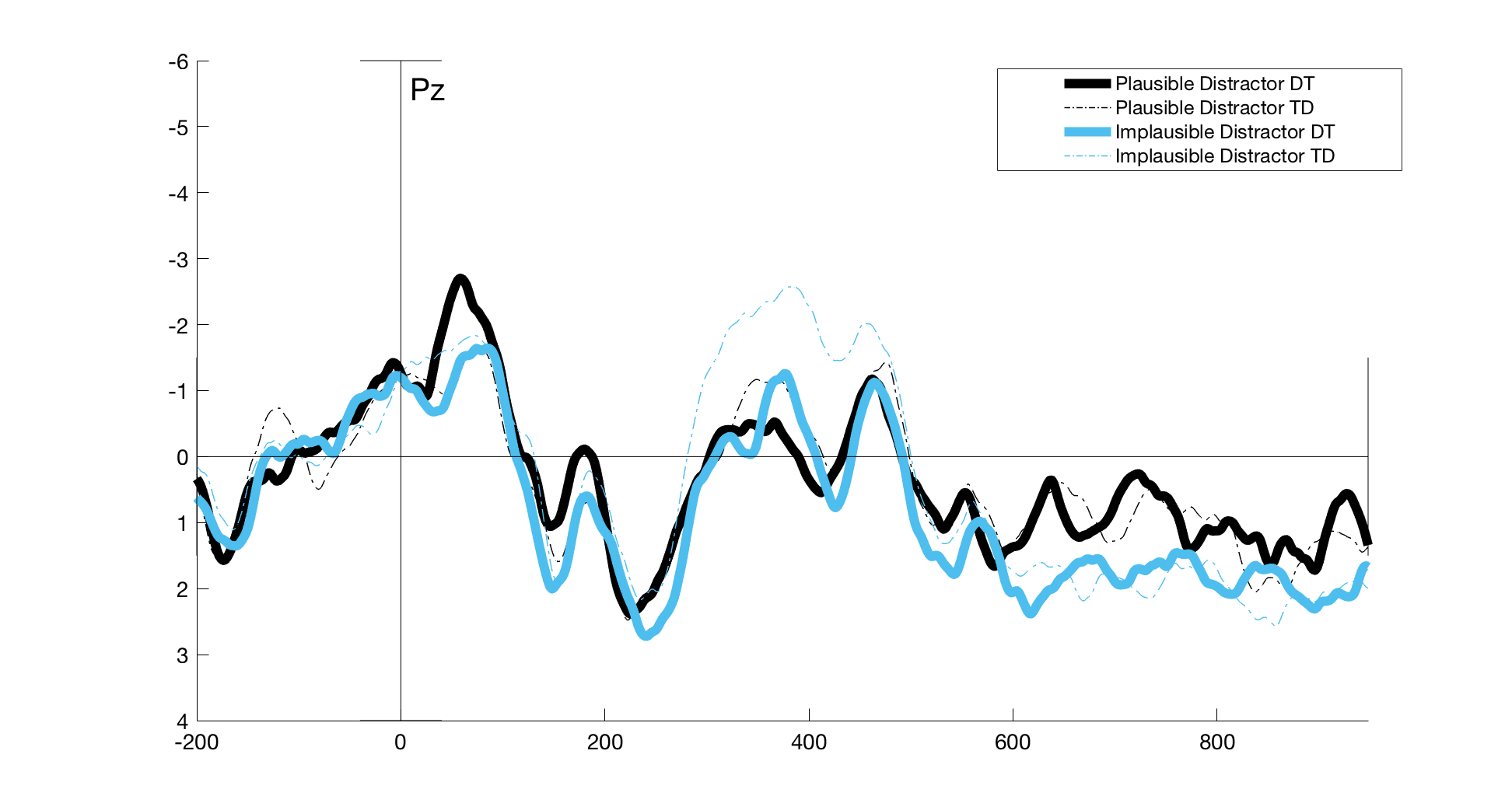

Supplement: Supplementary file 1 [file brainsci-09-00110-s001.zip › Supplementary_materials/S10_Pz.tif]

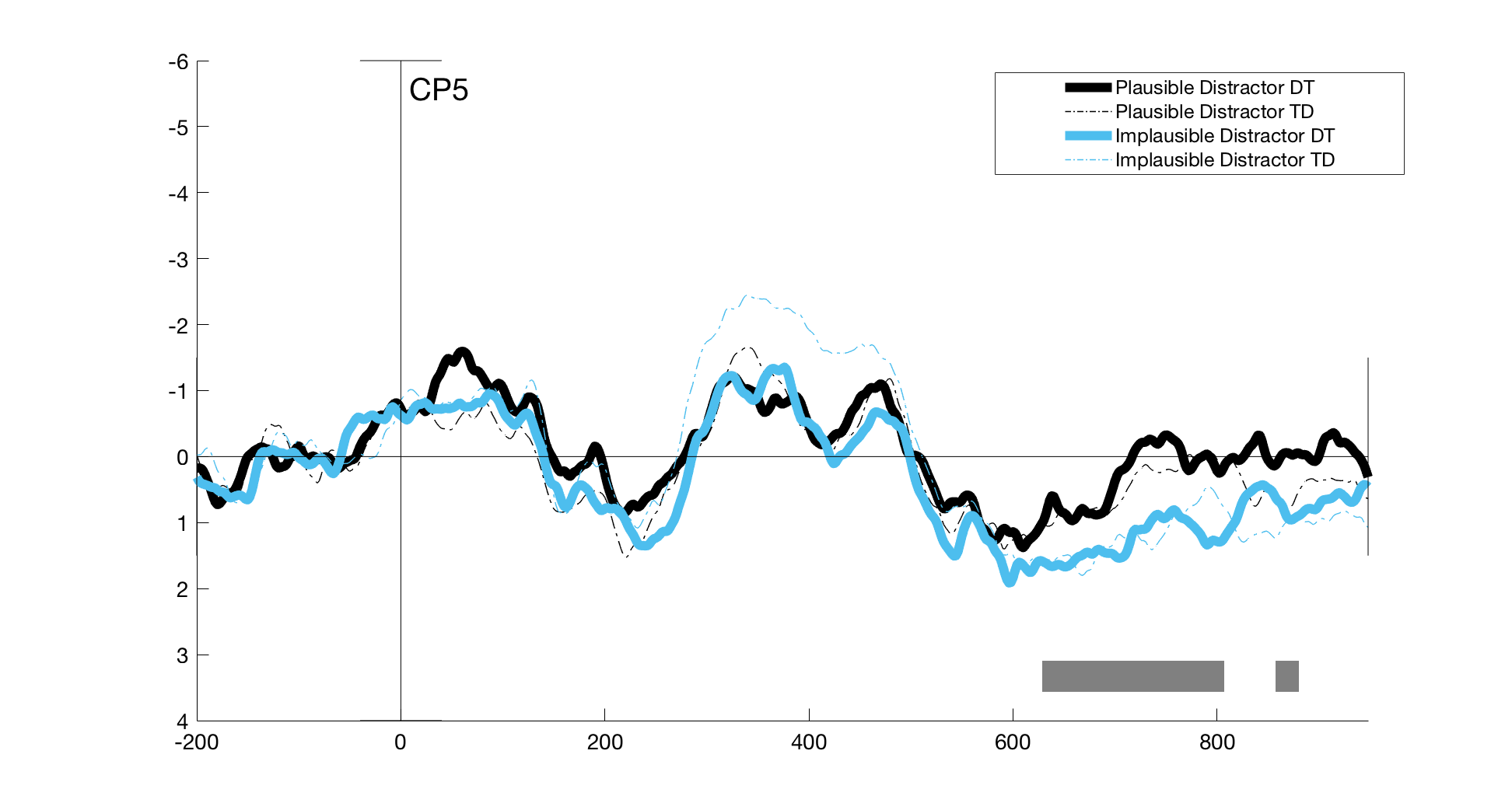

Supplement: Supplementary file 1 [file brainsci-09-00110-s001.zip › Supplementary_materials/S11_Cp5_biling.tif]

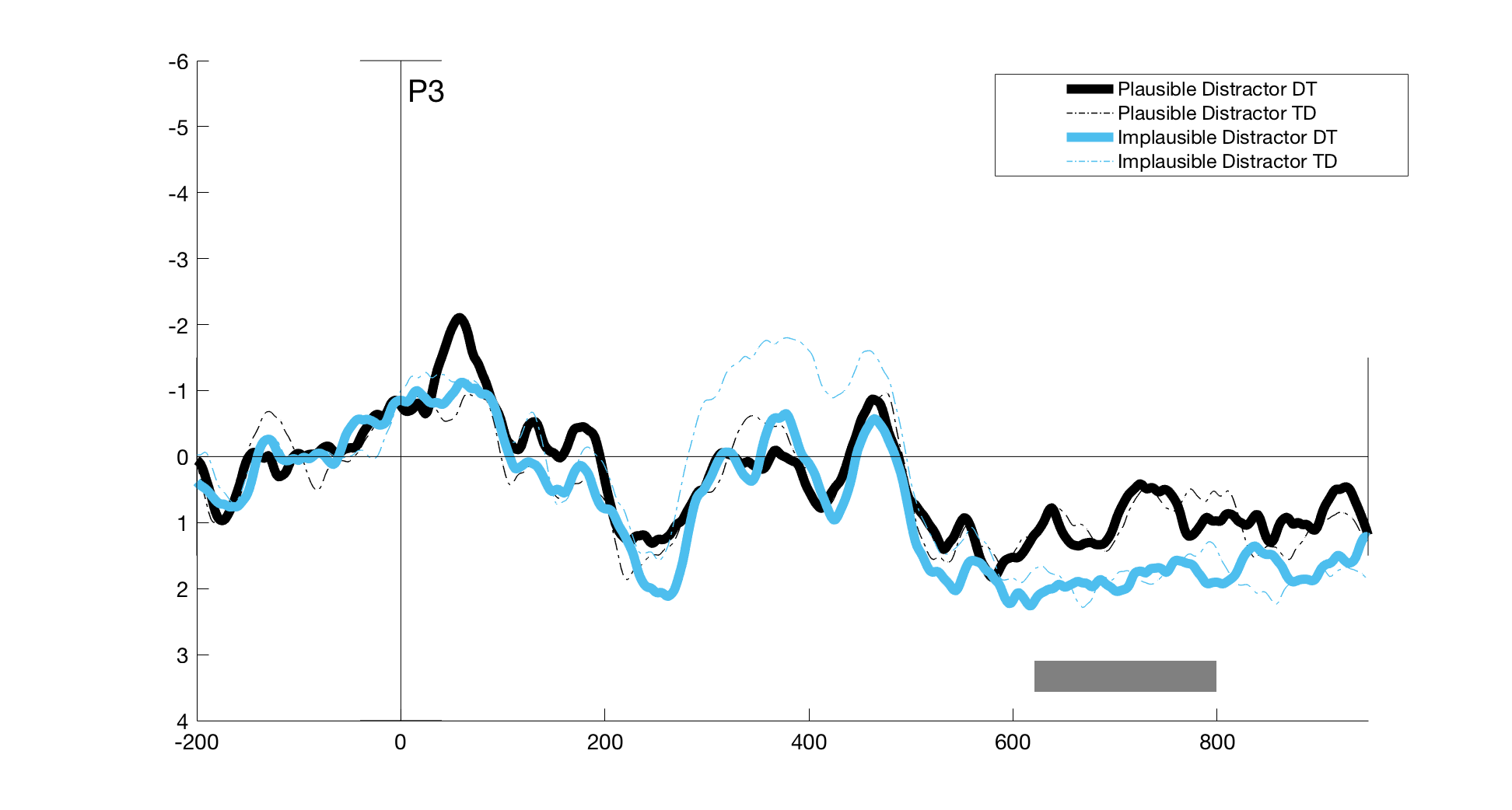

Supplement: Supplementary file 1 [file brainsci-09-00110-s001.zip › Supplementary_materials/S12_P3_biling.tif]

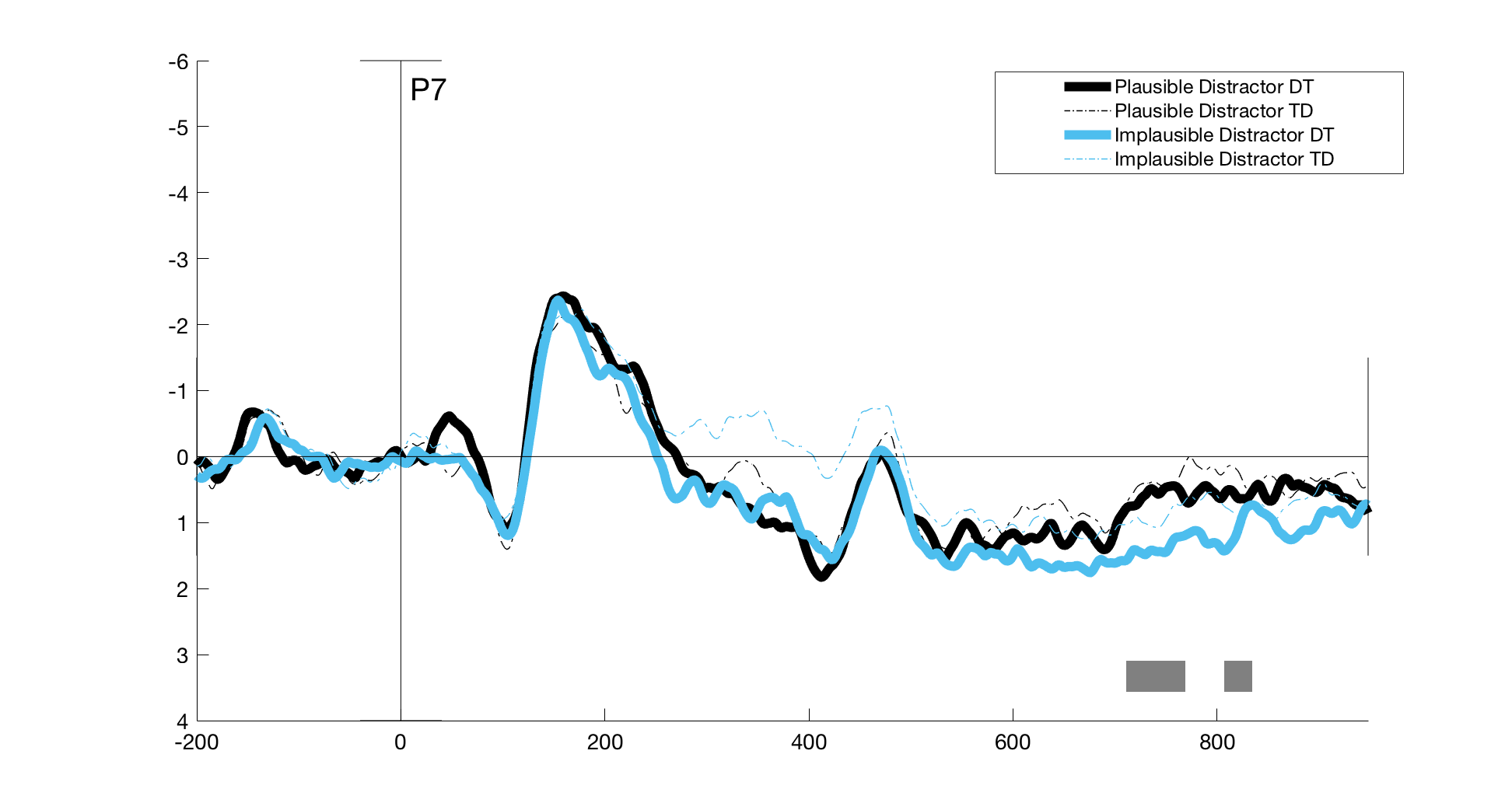

Supplement: Supplementary file 1 [file brainsci-09-00110-s001.zip › Supplementary_materials/S13_P7_biling.tif]

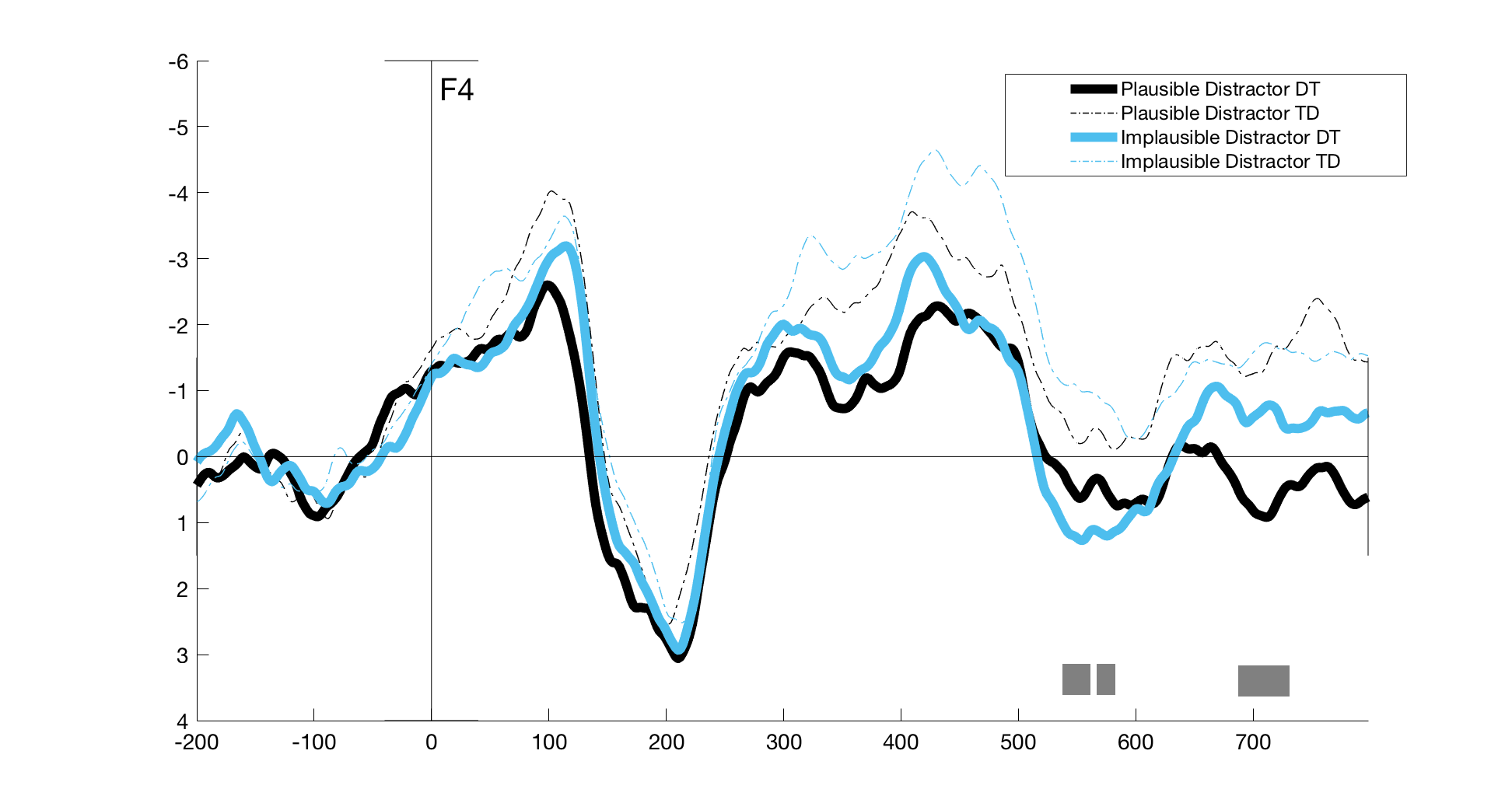

Supplement: Supplementary file 1 [file brainsci-09-00110-s001.zip › Supplementary_materials/S1_F4_mono.tif]

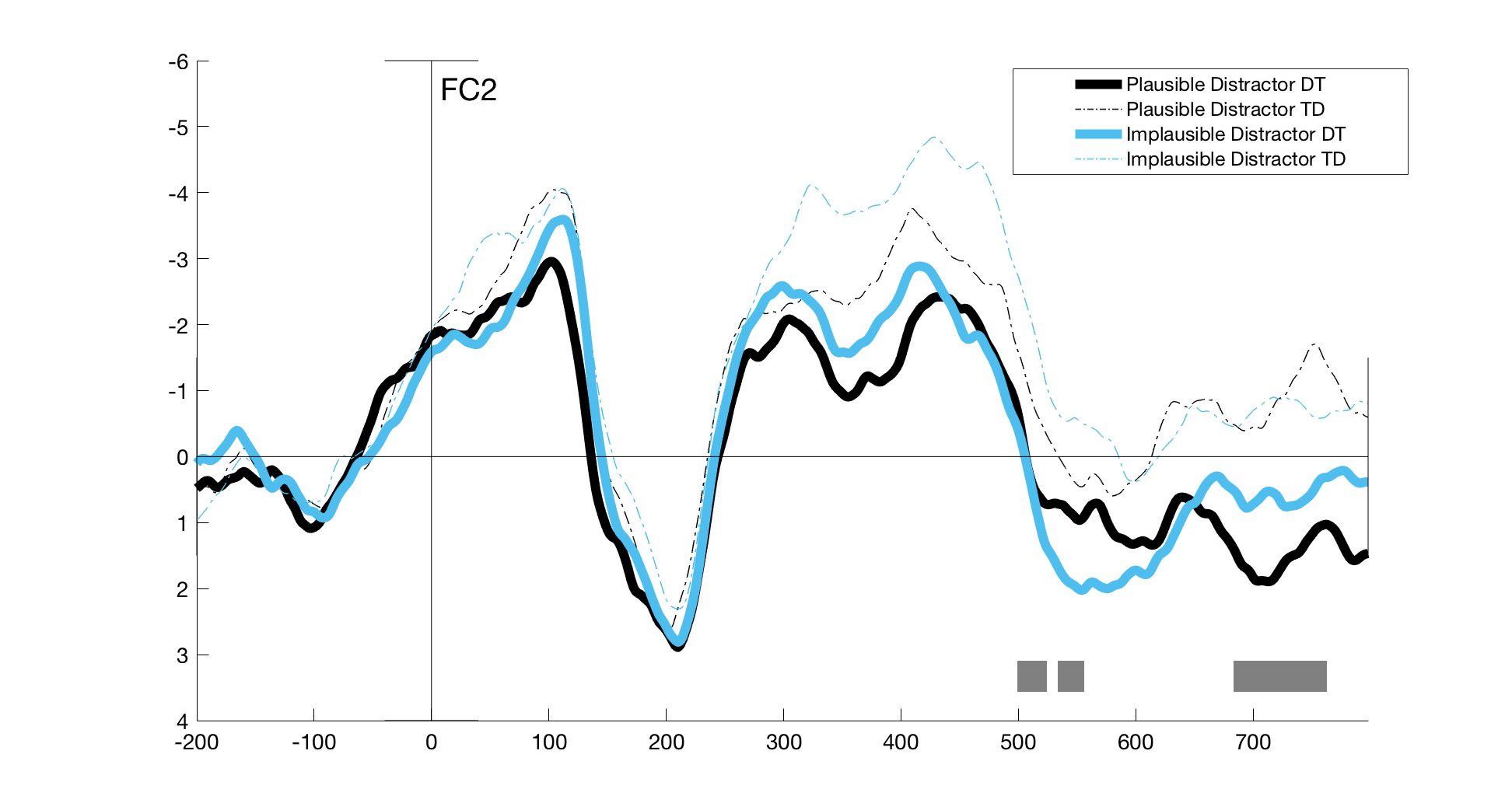

Supplement: Supplementary file 1 [file brainsci-09-00110-s001.zip › Supplementary_materials/S2_Fc2_mono.tif]

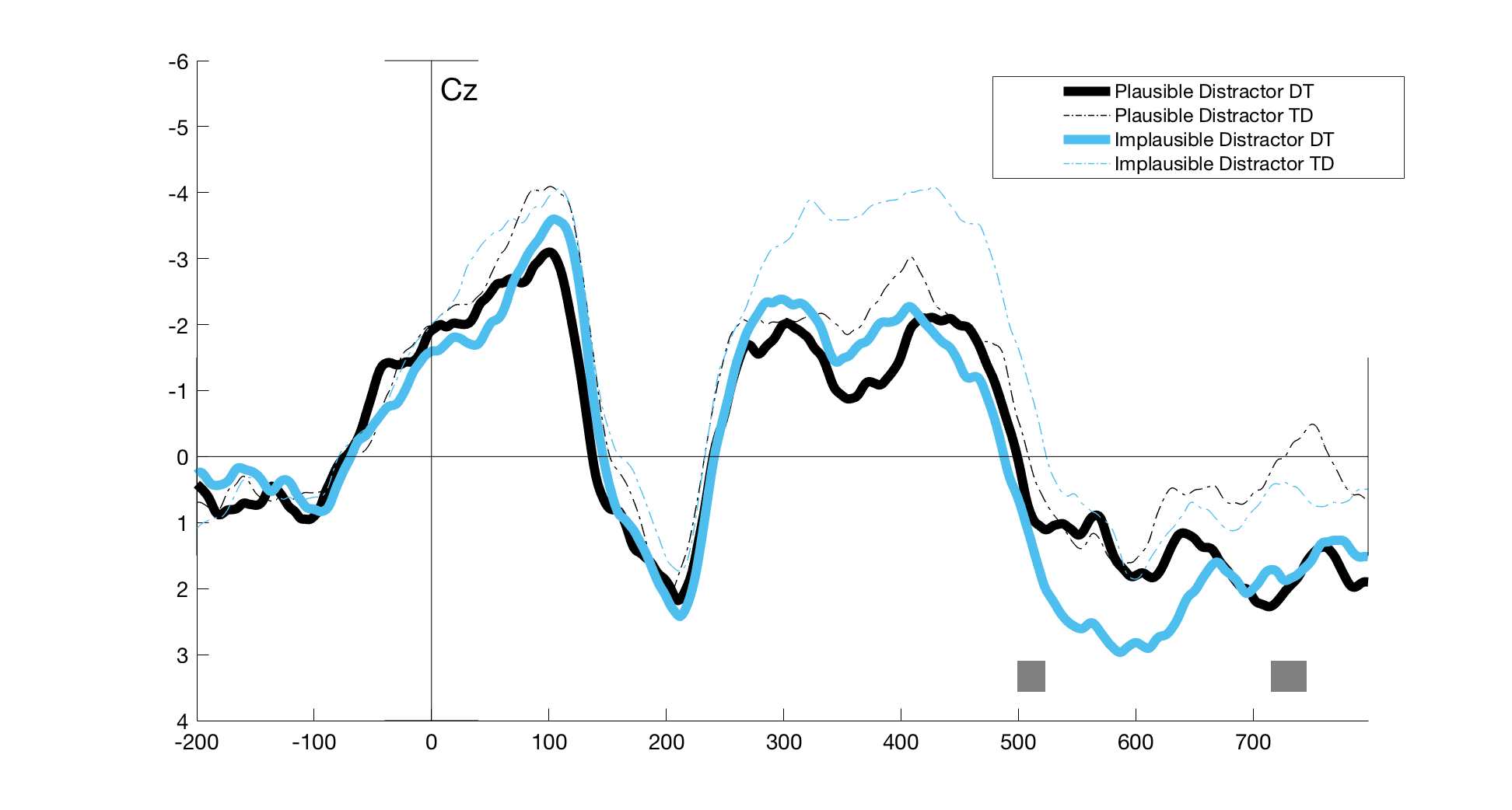

Supplement: Supplementary file 1 [file brainsci-09-00110-s001.zip › Supplementary_materials/S3_Cz_mono.tif]

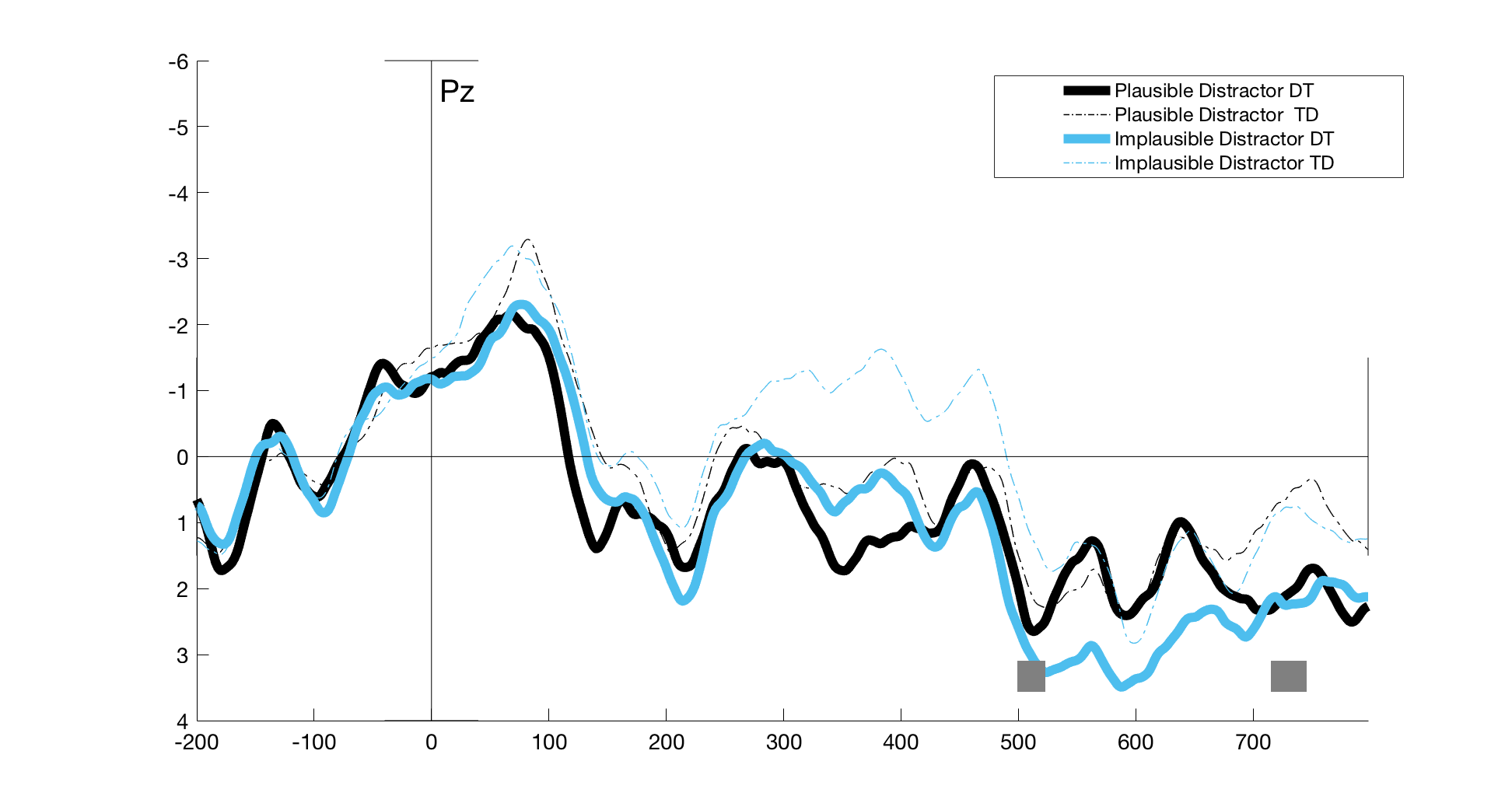

Supplement: Supplementary file 1 [file brainsci-09-00110-s001.zip › Supplementary_materials/S4_Pz_mono.tif]

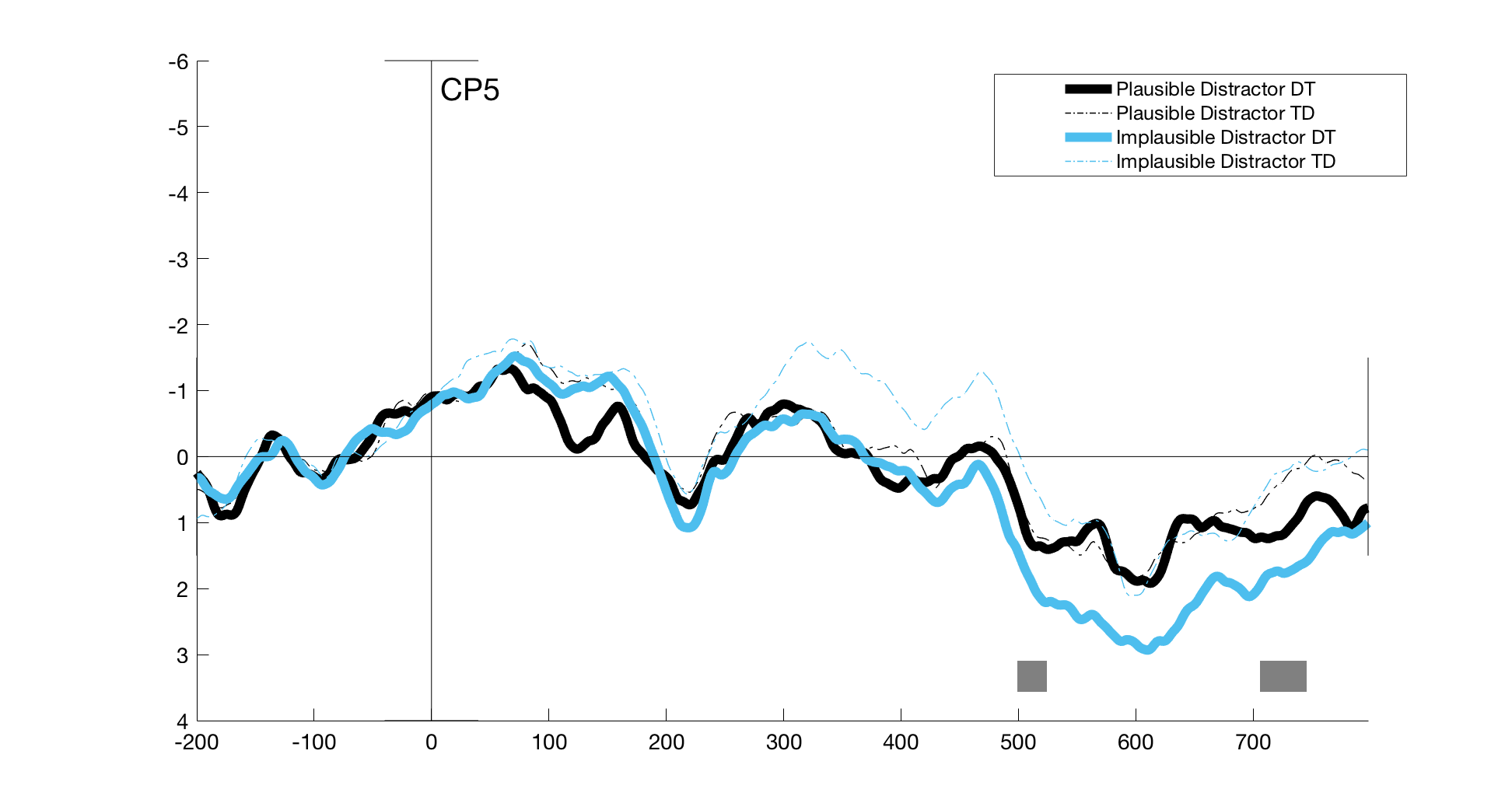

Supplement: Supplementary file 1 [file brainsci-09-00110-s001.zip › Supplementary_materials/S5_Cp5_mono.tif]

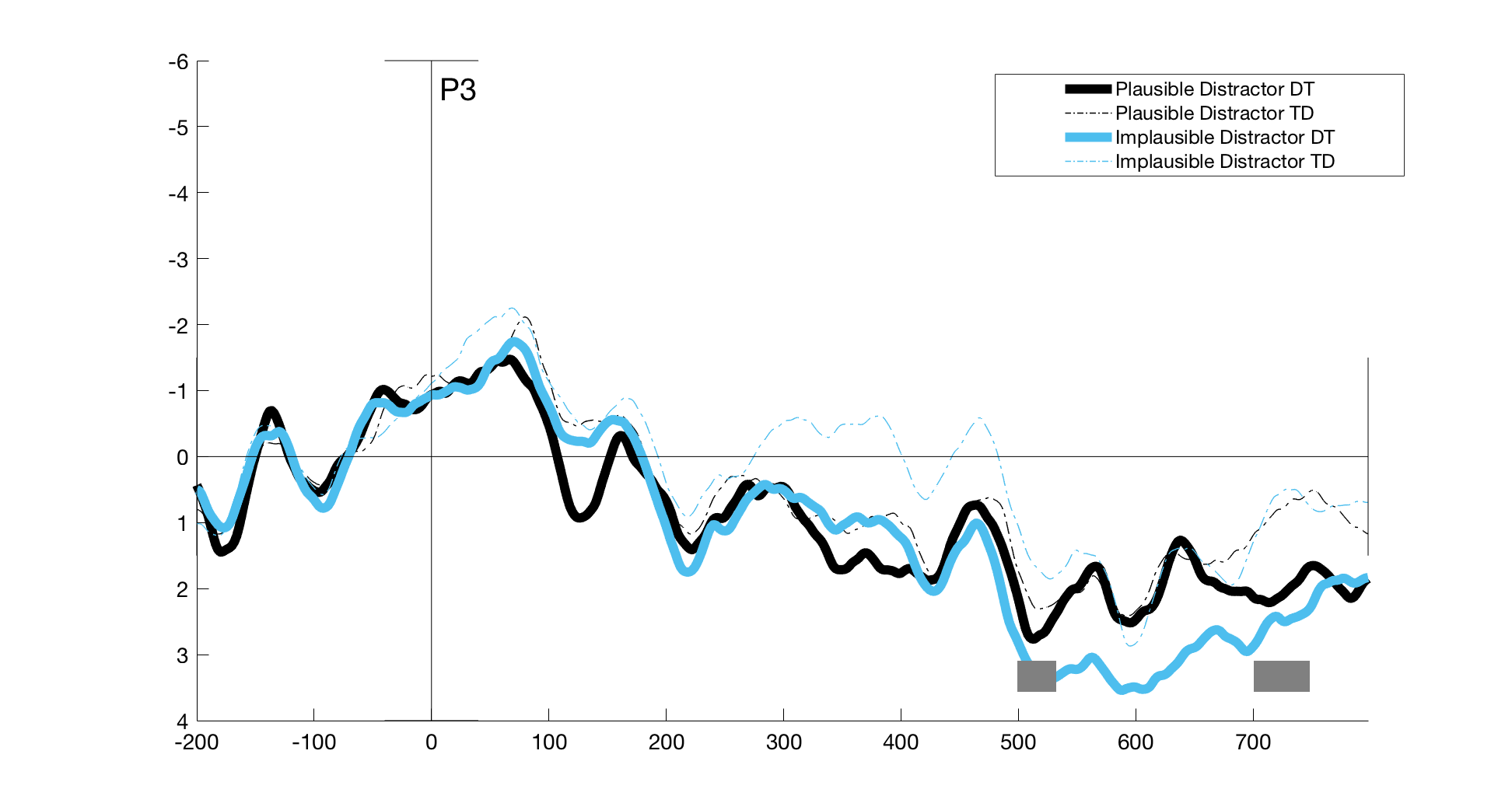

Supplement: Supplementary file 1 [file brainsci-09-00110-s001.zip › Supplementary_materials/S6_P3_mono.tif]

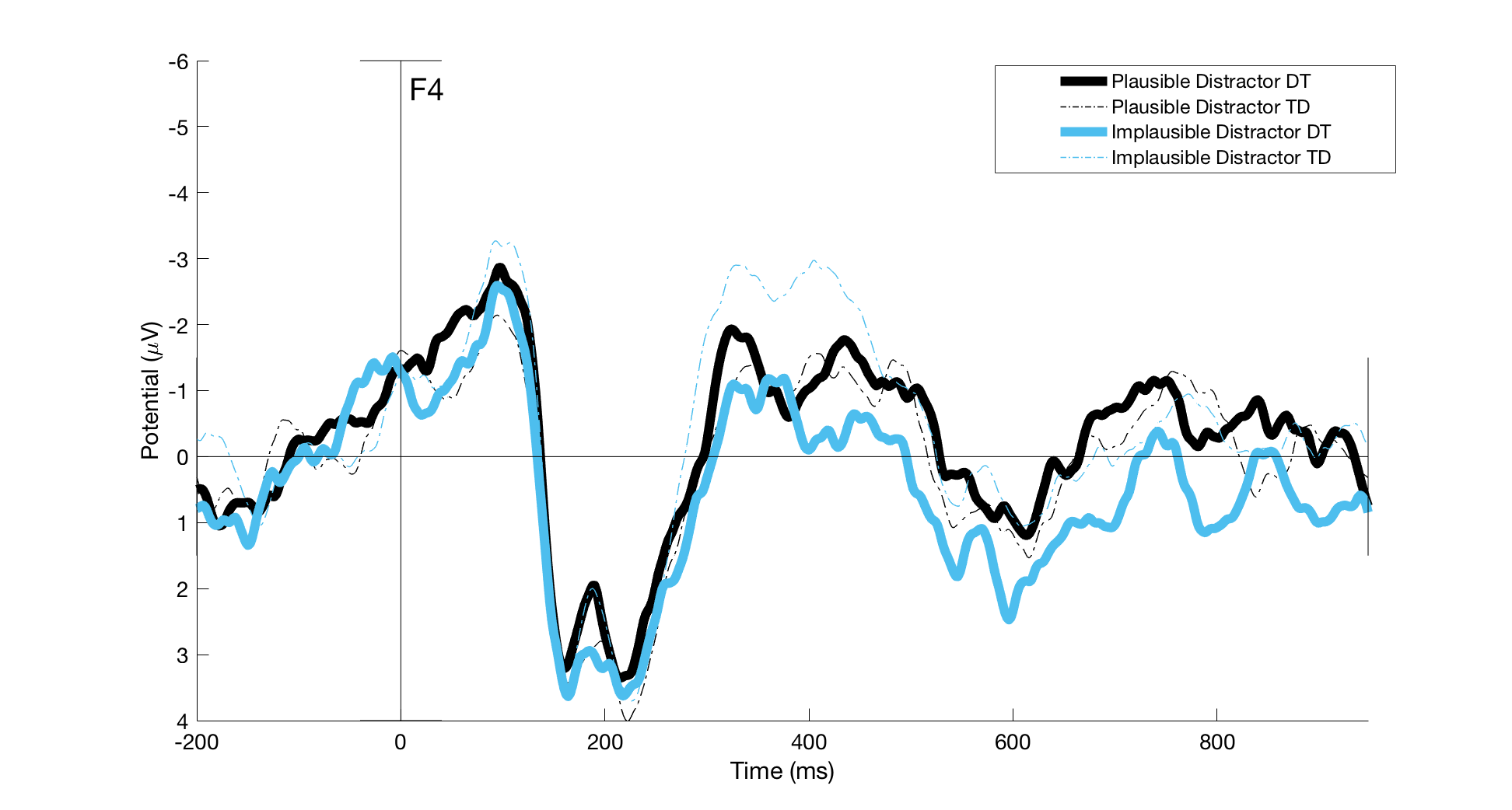

Supplement: Supplementary file 1 [file brainsci-09-00110-s001.zip › Supplementary_materials/S7_F4_biling.tif]

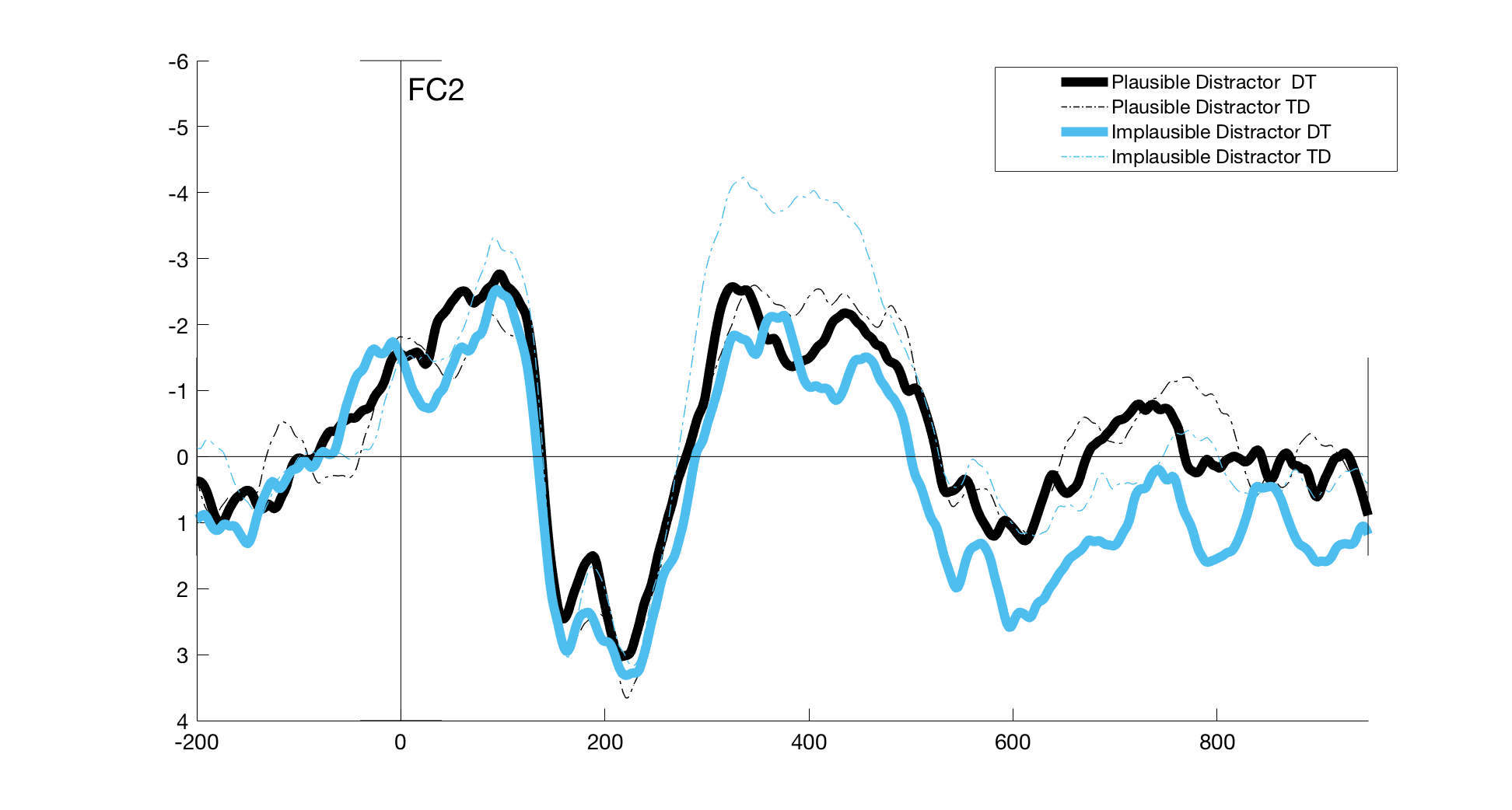

Supplement: Supplementary file 1 [file brainsci-09-00110-s001.zip › Supplementary_materials/S8_Fc2.tif]

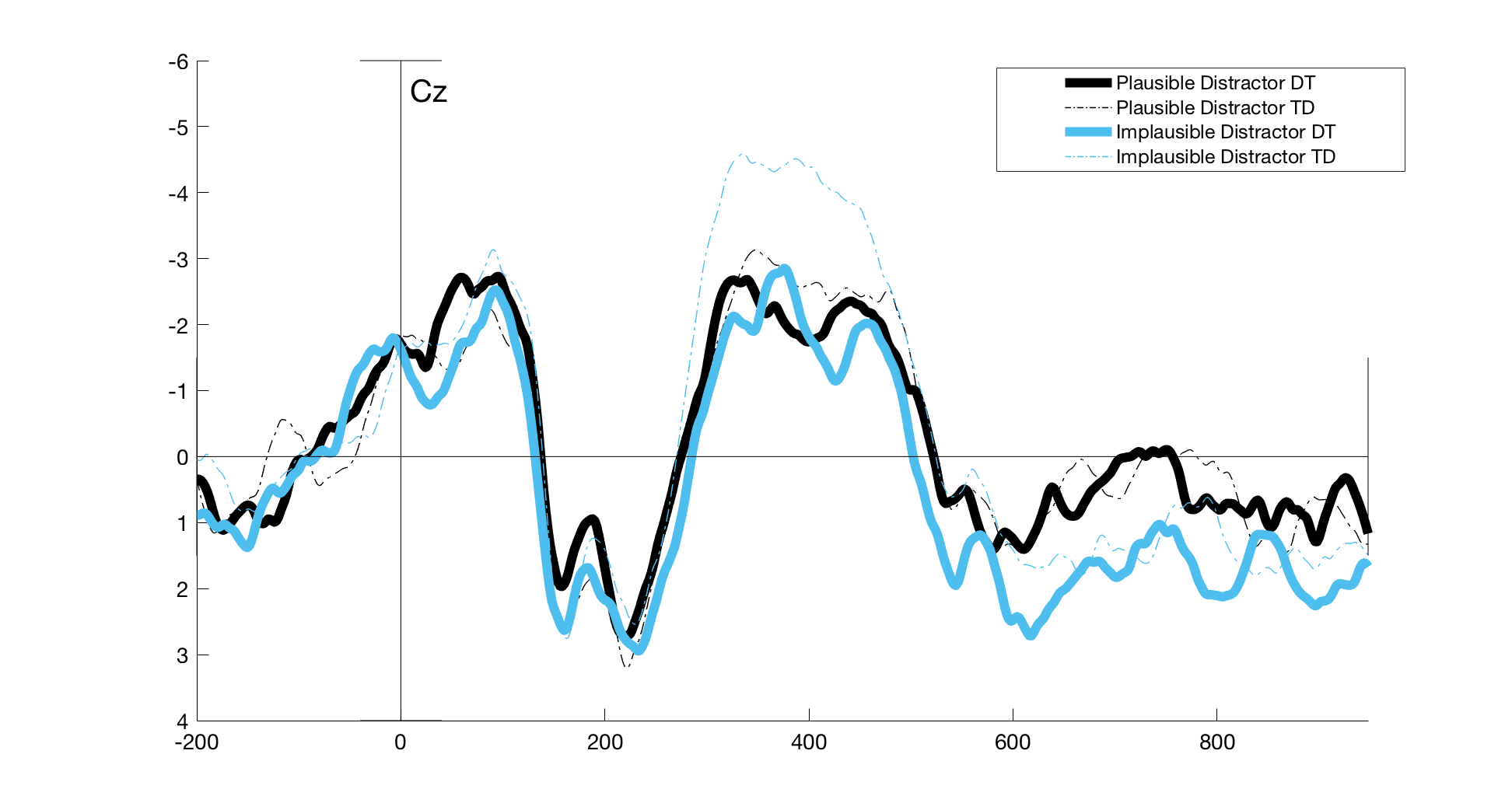

Supplement: Supplementary file 1 [file brainsci-09-00110-s001.zip › Supplementary_materials/S9_Cz_biling.tif]
